# Synergy in Immunostimulatory and Pro-Differentiation Effects of Vitamin D Analog and Fludarabine in Acute Myeloid Leukemias

**DOI:** 10.3390/cells14231841

**Published:** 2025-11-21

**Authors:** Subhradeep Haldar, Artem Petruk, Aleksandra Marchwicka, Andrzej Kutner, Monika Biernat, Dariusz Wołowiec, Ewa Marcinkowska

**Affiliations:** 1 Department of Medical Biotechnology, Faculty of Biotechnology, University of Wrocław, Joliot-Curie 14a, 50-383 Wrocław, Poland; subhradeep.haldar@uwr.edu.pl (S.H.); 335386@uwr.edu.pl (A.P.); aleksandra.marchwicka2@uwr.edu.pl (A.M.); 2 Department of Drug Chemistry, Pharmaceutical and Biomedical Analysis, Faculty of Pharmacy, Medical University of Warsaw, Banacha 1, 02-097 Warsaw, Poland; andrzej.kutner@wum.edu.pl; 3 Department of Hematology, Blood Neoplasms and Bone Marrow Transplantation, Wrocław Medical University, Pasteura 4, 50-367 Wrocław, Poland; monika.biernat@umed.wroc.pl (M.B.); dariusz.wolowiec@umw.edu.pl (D.W.)

**Keywords:** acute myeloid leukemia, differentiation, immune stimulation, 1,25-dihydroxyvitamin D analog, fludarabine

## Abstract

**Highlights:**

**What are the main findings?**

- We found that in leukemic cells combination of Fludarabine at a low concentration with 1,25-dihydroxyvitamin D analog synergistically regulate expression of many innate immunity-related and differentiation-related genes, which is followed by changes in respective protein levels.
- We found that in blasts from patients with myelodysplastic syndrome, 1,25-dihydroxyvitamin D analog induces myeloid differentiation.

**What are the implications of the main findings?**

- We propose to further investigate the combination of low concentration of Fludarabine with 1,25-dihydroxyvitamin D analog in relation to the subtypes of acute myeloid leukemia sensitive to this treatment.
- We propose to further investigate the efficacy of 1,25-dihydroxyvitamin D analog as a differentiation therapy for patients suffering from myelodysplastic syndrome.

**Abstract:**

Acute myeloid leukemia (AML) is an aggressive and often fatal hematopoietic malignancy, diagnosed predominantly in the elderly. The five-year survival of patients with AML is as low as 30%. Differentiation therapy of a subtype of AML, named acute promyelocytic leukemia (APL), using all-*trans* retinoic acid (ATRA) was the most successful example of a targeted therapy against AML. Epigenetic-based differentiation therapies for other subtypes of AML are also showing improvements in response and in survival rates. Thus, in this study, we investigated a potential differentiation therapy with a combination of 1,25-dihydroxyvitamin D (1,25D) analog (named PRI5202) and low concentration of Fludarabine. We show that such a combination elicits immunostimulatory and pro-differentiation effects in AML cells, specifically in those with activating mutations in fibroblast growth factor receptor (FGFR) and Janus kinase (JAK) pathways. We show here that both PRI5202 and Fludarabine are potent activators of the transcription of many innate immunity-related genes, and that, in combination, their effects are in many aspects synergistic. We propose that such a low-intensity regimen may be suitable for older patients with AML, who are unfit for intensive chemotherapy. We also present data indicating that PRI5202 induces myeloid differentiation in blasts from patients with myelodysplastic syndrome (MDS), and we propose to further investigate PRI5202 as a differentiation therapy for patients suffering from MDS.

## 1. Introduction

Acute myeloid leukemias (AML) are diseases of the myeloid lineage of hematopoiesis. They constitute a broad family of malignancies, which have various molecular backgrounds. AMLs are characteristic of older people, with the median age at diagnosis of 68 [1]. The global incidence of AMLs has been increasing in recent years, which has been attributed to the growing longevity [2]. Because AML often presents with life-threatening manifestations, the timely diagnosis and fast initiation of chemotherapy are of primary importance. However, not always treatment with the curative intent is possible, because such therapy is very intensive. Thus, before the chemotherapy is initiated, an assessment of the patient’s fitness is required. The standard treatment for younger patients is intensive chemotherapy with the intention of achieving complete remission of the disease, followed by a bone marrow transplantation [1]. Usually, the intensive chemotherapy is not recommended for patients older than 75, but additional measures, such as comorbidities or general performance, are taken into consideration [3]. For patients ineligible for intensive chemotherapy, less intensive regimens are employed. Outcomes for such patients have improved in recent years, particularly for those harboring nucleophosmin (*NPM1*) or isocitrate dehydrogenase 2 (*IDH2*) gene mutations in their blast cells [4].

Usually, the best results in hematological malignancies are obtained when therapies target proteins resulting from mutated genes [5]. The greatest success was the introduction of all-*trans* retinoic acid (ATRA) to treat a subtype of AML, acute promyelocytic leukemia (APL), where the retinoic acid receptor α gene (*RARA*) is fused to the promyelocytic leukemia gene (*PML*) due to a chromosomal translocation [6]. When first described in 1957, APL was considered “the most malignant form of acute leukemia” [7], while nowadays, APL can be successfully treated with the combination of ATRA, arsenic trioxide (ATO) and chemotherapy [8,9]. ATRA induces differentiation of APL blasts towards granulocytes, which lose their immortality following the differentiation process [10]. Unfortunately, ATRA is not effective in other subtypes of AML and can even be harmful in some cases [11]. Another type of less aggressive therapy for AML targets epigenetics and is suitable for older patients when combined with the B-cell lymphoma 2 protein (Bcl-2) inhibitor, venetoclax [12].

A differentiation effect of the physiologically active form of vitamin D, 1,25-dihydroxyvitamin D (1,25D), towards AML blasts was well documented in vitro and in vivo [13]. 1,25D is a ligand to the nuclear vitamin D receptor (VDR), which acts as a ligand-activated transcription factor [14]. However, supraphysiological concentrations of 1,25D can cause hypercalcemia in humans. To overcome this limitation, semi-selective analogs of 1,25D have been developed, some of which are already in clinical use for the treatment of psoriasis and secondary hyperparathyroidism [15,16]. Despite the tremendous effects of vitamin D analogs against AML blasts in vitro, they are not in clinical use, most probably due to the lack of precisely defined subtypes of AML responding to such treatment [17]. One possible reason may be that not all subtypes of AML express the *VDR* gene and, therefore, lack the target protein for 1,25D or its analogs. In our previous study, we documented that AML cells with constitutively active fusion kinase, fibroblast growth factor receptor (FGFR) Oncogene Partner 2–fibroblast growth factor receptor 1 (FOP2-FGFR1), do not express *VDR*, while after disruption of the fusion gene, they do [18]. The fusion kinase FOP2-FGFR1 was documented to increase the activation of signal transducer and activator of transcription (STAT) transcription factors in AML cells [18,19]. On the other hand, AML cells with overexpression of wild-type (wt) FGFRs have high expression of *VDR* and respond to 1,25D very effectively [20]. A recent study identified the AML cells with isocitrate dehydrogenase gene (*IDH*)-R132H mutation as good responders to 1,25D due to the higher levels of VDR protein than in the cells with wt-*IDH* [21]. It thus seems plausible that combinations of vitamin D analogs with either inhibitors or activators of certain signal transduction pathways may become an effective differentiation therapy for some patients with AML. Moreover, due to the immunostimulatory effects of 1,25D, specifically in the aspect of innate immunity, vitamin D analogs are promising agents for combination anti-AML treatments [22,23,24].

The above-mentioned over-activation of STAT transcription factors is relatively common in AMLs. The mechanisms may be various, such as mutations of the genes encoding upstream transmembrane receptors, mutations in Janus kinase (JAK) genes, in the STATs themselves, or due to the autocrine/paracrine cytokine production [25]. A recent report documented that high expression of *STAT1* promotes survival of leukemic stem cells [26]. Since JAKs and STATs are druggable, there were numerous trials of inhibitors of JAK1, JAK2, STAT3, and STAT5, and some of them were promising when used in combination treatments [27]. However, in our previous research, we observed that the best pro-differentiating effect was obtained when STAT1 inhibitor, Fludarabine, was used either alone or in combination with 1,25D [20].

Fludarabine belongs to the class of antimetabolite drugs, which are similar in structure to the purine and pyrimidine bases, and disrupt DNA or RNA synthesis either by being incorporated into the nucleic acid or by blocking enzymes essential for nucleic acid synthesis. Fludarabine is a well-known fluorinated purine analog, administered as a prodrug, Fludarabine phosphate, which is dephosphorylated in the bloodstream, and taken up as 9-beta-D-arabinosyl-2-fluoradenine by the cells. Inside the cells, it is re-phosphorylated to 2-fluoro-ara-ATP, which competes with natural deoxyadenosine triphosphate to incorporate into the DNA and terminates its elongation. 2-Fluoro-ara-ATP competes with the natural deoxynucleotide, directly inhibiting DNA polymerases and DNA primase, which synthesizes an RNA primer required for the initiation of new strand synthesis. 2-Fluoro-ara-ATP is also an effective inhibitor of ribonucleotide reductase, resulting in a lowering of cellular deoxynucleotide pools [28]. Fludarabine is used in younger AML patients who relapse after initial complete remission, as a part of salvage therapies named FLAG (Fludarabine, cytarabine, granulocyte colony-stimulating factor), sometimes combined with idarubicin (FLAG-IDA) [29,30]. Fludarabine has been used in high-risk myelodysplastic syndrome (MDS) [29] or in intensive myeloablative regimens before stem cell transplantations in AML [31,32], and is included in the leading therapies against chronic lymphocytic leukemia [33]. However, these intensive chemotherapies are not suitable for less fit and old patients. It was reported that, at low concentrations, Fludarabine inhibits STAT1, but unfortunately, the exact mechanism of this inhibition is not understood [34,35].

Therefore, in our current study we decided to test a combination of vitamin D analog and Fludarabine against AML and MDS cells. In our previous work, we evaluated a panel of semi-selective vitamin D analogs to identify compounds that induce stronger differentiation than 1,25D, while causing less hypercalcemia. One such analog is PRI5202, which binds to VDR with affinity similar to 1,25D, but is more potent in inducing cell differentiation, and less calcemic than 1,25D [36]. PRI5202 has shown efficacy against AML in mice [37] and against ovarian cancer cells in vitro [38]. In the current study, we employed AML cell lines with varying sensitivity to 1,25D-induced differentiation, as well as primary blasts from patients, to assess whether the combination of PRI5202 and Fludarabine produces synergistic pro-differentiation and immunomodulatory effects.

## 2. Materials and Methods

### 2.1. Cell Lines and Bone Marrow Cells

U937 cells were purchased from the German Resource Center for Biological Material (DSMZ GmbH, Braunschweig, Germany) [20], HL60 cells were from the cell bank at the Institute of Immunology and Experimental Therapy in Wrocław (Poland) [36], and HEL cells were a kind gift from Prof. Aleksander Czogalla from our faculty [39]. The cells were cultured in RPMI1640 medium (Biowest, Nuaillé, France) with 10% FBS, 2 mM L-glutamine, 100 units/mL penicillin, and 100 μg/mL streptomycin (all from Sigma-Aldrich, St. Louis, MO, USA) and maintained at standard cell culture conditions. U937 cells were transfected using the pcDNA(TM)3.1(+) plasmid from Thermo Fisher Scientific Inc. (Worcester, MA, USA), either empty or encoding FGF receptors 1 and 2, as described before [20]. Transfected cells were cultured in the medium supplemented with geneticin at a concentration of 1 μg/mL.

Human bone marrow samples were obtained from the patients of the Department of Hematology, Blood Neoplasms, and Bone Marrow Transplantation, all of whom gave informed consent for this study. The study was accepted by the local Ethical Committee (permit No KB 401/2023N from 23 November 2023). Approximately 6 mL of bone marrow were diluted with phosphate-buffered saline (PBS) in a 1:1 ratio, carefully layered onto an equal volume of Lymphosep (Biowest), and centrifuged at 400× *g* for 30 min at room temperature. The opaque interface was transferred into a fresh sterile tube and washed three times with PBS. Then, mononuclear cells were moved to Marrow Prime Complete Medium (Capricorn Scientific GmbH, Ebsdorfergrund, Germany) and maintained at standard cell culture conditions.

### 2.2. Reagents

PRI5202 was synthesized at the Pharmaceutical Research Institute (Warsaw, Poland). The compound was aliquoted and stored in glass ampoules under argon at −20 °C. The compound was dissolved in an absolute ethanol from Carl Roth GmbH (Karlsruhe, Germany) to 100 µM and subsequently diluted in the culture medium to the required concentration. Fludarabine was purchased from MedChemExpress (Monmouth Junction, New Jersey, NJ, USA) and was resuspended in dimethyl sulphoxide (DMSO; Bioshop, Burlington, ON, Canada) to 10 mM and stored at −20 °C. Further dilutions of Fludarabine were in the cell culture medium. IFNγ was from ImmunoTools GmbH (Friesoythe, Germany) and resuspended to 100 µg/mL in sterile water and stored at −20 °C. NaCl and HCl were from Carl Roth GmbH (Karlsruhe, Germany). Tris base, SDS, Glycine, Ammonium Persulfate, and TEMED (N,N,N’,N’-tetramethylethylenediamine) were purchased from Bioshop (Gloucester, MA, USA). EDTA and EGTA were from Thermo Fisher Scientific Inc. Triton™ X-100, Glycerol, β-mercaptoethanol, and Bromophenol Blue were from Sigma-Aldrich. A 40% Acrylamide solution was obtained from AppliChem GmbH (Darmstadt, Germany).

### 2.3. Sequencing of mRNA and Analysis of the Results

Single samples from all U937 sublines, HL60, and HEL cell lines (7.5 × 10^6^ cells/sample) were exposed for 48 h to 10 nM PRI5202 or/and 315 nM Fludarabine (or 0.1% EtOH as a vehicle control). Post-exposure, total RNA was isolated using Tri Reagent (Sigma) as per the manufacturer’s recommendation. The RNA quantity and quality were assessed by NanoDrop 2000 (Thermo Fisher Scientific Inc.) and 4200 TapeStation system (Agilent, Santa Clara, CA, USA). Strand-specific mRNA sequencing on the Illumina platform was performed by Novogene (Cambridge, UK), where directional mRNA library preparation, Illumina sequencing, and the raw RNA read mapping were performed. The raw read counts of the mapped genes across samples were calculated by Novogene, and further bioinformatic analyses were performed in NovoMagic platform. To do so, EdgeR package v. 3.22.5 included with NovoMagic software was used to produce the list of the differentially expressed genes (DEGs) and the related data for the comparison between two chosen samples at a time. The raw read count data per gene in the two concerned samples were normalized using the trimmed mean of M values (TMM) method to correct for sequencing depth [40]. Following that, *p*-value estimation for hypothesis testing of the differential expression was performed using exact test (https://www.bioconductor.org/packages//2.7/bioc/vignettes/edgeR/inst/doc/edgeR.pdf, accessed on 12 November 2025). Finally, adjusted *p*-values for the false discovery rate (FDR) values of multiple hypothesis testing were calculated. Further, the expression levels of the genes across drug treatment samples were compared to the vehicle treatment. The obtained list was filtered for the gene expression using a threshold of |Log_2_FoldChange| of 1 and adjusted *p*-value < 0.05. All DEGs and their normalized expression values were identified using EdgeR package. The list was finally filtered for protein-coding genes only. To assess the regulation of gene expression as synergistic or not, the common upregulated DEGs across cell lines were merged using PowerQuery tool v. 2.56.5023.1181 from Microsoft (Washington, DC, USA), with their Log_2_FoldChange values, and were categorized as synergistic or not (https://en.wikipedia.org/wiki/Additive_effect#cite_note-21; accessed on 26 August 2025), using the equation:

(1) $$Synergy\;if\;Antilog2FC\;\left(Combi\right)>Antilog2FC\;\left(PRI5202\right)+Antilog2FC\;\left(Fludarabine\right)$$

In cases of downregulated DEGs, inverse absolute values were considered.

To rank the genes in terms of the effectiveness of the combination treatment, synergy scores of each gene were computed using the equation:

(2) $$Synergy\;score=Antilog2FC\left(Combi\right)-(Antilog2FC\left(PRI5202\right)+Antilog2FC\left(Fludarabine\right))$$

### 2.4. Reverse Transcription Quantitative Polymerase Chain Reaction (RT-qPCR)

Post 48 h of exposure of 7.5 × 10^6^ cells/sample to 10 nM PRI5202, 315 nM Fludarabine, or combination (or 0.1% EtOH as a vehicle), total RNA was isolated using Tri Reagent (Sigma-Aldrich), Fenozol (A&A Biotechnology), or Extrazol (QIAGEN; Hilden, Germany), as per manufacturers’ recommendations. RNA quantity and quality were determined using Nanodrop 2000 (Thermo Fisher Scientific Inc.). In total, 2 µg of RNA was transcribed into cDNA using High-Capacity cDNA Reverse Transcription kit (Applied Biosystems, Foster City, CA, USA). RT-qPCR reaction was performed using SensiFAST SYBR Hi-ROX (Bioline, London, UK) and CFX Real-time PCR System (Bio-Rad Laboratories Inc., Hercules, CA, USA). The primers used for RT-qPCR are listed in Supplementary Table S1 [20,41,42]. A total of 20 ng of cDNA and 100 nM primers were diluted in 1X SYBR green master mix and water, then the following protocol was used for the RT-qPCR reactions: thermal activation of polymerase (95 °C for 2 min), 40 cycles of denaturation (95 °C for 5 s), annealing of primers (primer-specific temperature for 10 s), extension of the complimentary DNA strands (72 °C for 5 s), and quantification, followed by a melt curve gradient of 60 °C to 95 °C with 0.5 °C increment for 15 sec and quantification. Baseline threshold values for each quantification per cell line were determined from the primer efficiency experiments with serially diluted cDNA. The gene expression within each sample was normalized to *GAPDH*, and the fold change per treatment was quantified in relation to vehicle-treated samples following the 2^−ΔΔCt^ method. All relative quantified RT-qPCR data are shown as Log_2_Fold values.

### 2.5. Flow Cytometry

For cell lines, 5 × 10^5^ cells/sample were used, and for donor-derived mononuclear cells, 1 × 10^6^ cells/sample were used. After 96 h exposure of the cells to 10 nM PRI5202, 315 nM Fludarabine, or their combination (or 0.1% EtOH as a vehicle), the cells were harvested and washed with ice-cold phosphate-buffered saline (PBS) supplemented with 0.1% bovine serum albumin (BSA) from Santa Cruz Biotechnology, Inc. (Santa Cruz, CA, USA). The cells were incubated with antibodies for 45 min on ice. Donor-derived mononuclear cells were additionally stained with 7-AAD viability dye (1:20) from BioLegend (San Diego, CA, USA) for the last 15 min of incubation. The mouse-anti CD11b-FITC (1:25), mouse-anti CD14-PE (1:50), mouse-anti CD150-AF488 (1:25), mouse-anti CD354-APC (1:25), and matching isotype controls (equal dilution as respective antibody) were from BioLegend. The cells were then washed and resuspended in 350 μL of PBS/0.1% BSA prior to analysis. For studies of apoptosis and necrosis, APC-Annexin V apoptosis detection kit was used according to the manufacturer’s instruction (BioLegend). All samples were analyzed on the Becton Dickinson Accuri C6 (San Jose, CA, USA). Data analysis was performed using Becton Dickinson Accuri C6 v. 1.0.246.21 software.

### 2.6. Western Blotting

To study VDR, 5 × 10^6^ cells/sample were exposed to 10 nM PRI5202, 315 nM Fludarabine, or a combination (or vehicle) for 24 h. To study STAT1 and pSTAT1, 5 × 10^6^ cells/sample were incubated with 315 nM Fludarabine (or vehicle) for 24 h prior to treating them with 100 ng/mL IFNγ for 15 min. Then the cells were washed with PBS and lysed using NE-PER Nuclear and Cytoplasmic Extraction Reagents (Thermo Fisher Scientific Inc.) according to the user’s manual. Obtained lysates were denatured by adding 5× sample buffer (50 mM Tris-HCl, 12.5% SDS, 40% (*v*/*v*) glycerol, 25% (*v*/*v*) β-mercaptoethanol, 0.05% Bromophenol Blue) and boiling at 98 °C for 5 min. For Western blotting, 25 μL of lysates and 5 μL of Perfect^™^ Tricolor Protein Ladder from EURx Sp. z o.o. (Gdańsk, Poland) were separated on 12% SDS-PAGE gels and transferred to PVDF membranes (Merck, Darmstadt, Germany). The membranes were then dried overnight and blocked with TBST blocking buffer (0.8% NaCl, 20 mM Tris-HCl; pH 7.6 with 0.05% (*v*/*v*) Tween 20) supplemented with 1× Casein Blocking Buffer (Sigma-Aldrich) for 30 min. Then the membranes were incubated with primary antibodies (mouse anti-actin, sc-8432, 1:100, 2 h, RT; mouse anti-HDAC2, sc-9959, 1:200, 1.5 h, RT; mouse anti-VDR, sc-13133, 1:250, 2.5 h, RT; mouse anti-STAT1, sc-464 and mouse anti-pSTAT1, sc-8394, both 1:100, overnight at 4 °C; all from Santa Cruz Biotechnology) and a horseradish peroxidase-conjugated secondary antibody (goat anti-mouse IgG (H + L) 1:5000, 1 h, RT) from Jackson Immunoresearch (West Groove, PA, USA). All primary and secondary antibodies were diluted in TBST blocking buffer. The membranes were washed with TBST buffer (3 times for 5 min) before exchanging the antibodies and before imaging. The protein bands were visualized with a Western blotting luminol reagent from Santa Cruz Biotechnology and imaged with the ChemiDoc XRS+ system from Bio-Rad. Then the membranes were stripped with SDS buffer (0.125 M Tris, 0.1 M glycine, 0.5% SDS), dried again, and probed with subsequent antibodies.

In order to quantify the protein levels, the intensity of each band was measured using Image Lab V6.0.1 software from Bio-Rad. For VDR level quantification, the intensities of VDR protein bands in cytosolic fraction samples were normalized to the intensities of corresponding actin bands, while in nuclear fraction samples, they were normalized to the intensities of corresponding HDAC2 bands. Considering the normalized intensity in vehicle-treated samples as 100% of the VDR protein level, relative protein levels were calculated for all other treatment groups.

### 2.7. Statistical Analysis and Figure Preparation

At least three independent biological replicates were performed for all experiments, comprising cell lines, except for the mRNA-sequencing (RNAseq) experiment. The statistical analyses were performed using GraphPad Prism 10 software (GraphPad Software, Boston, MS, USA). After identifying outliers in the data using the ROUT method, Brown-Forsythe and Barlett’s test for equality in group variance was performed. Sample groups without significantly different variance were proceeded with ordinary one-way ANOVA coupled with Šidák’s multiple comparison test. The remaining groups were analyzed using the Welch-ANOVA test, coupled with Dunnet’s T3 test with multiple comparisons. The aforementioned ad hoc multiple comparison test was performed to compare the statistical significance between single drug treatments and combination treatment. The computed *p*-values are represented as * (<0.033); ** (<0.002); and *** (<0.001). In order to calculate the statistically significant synergistic effects, an F-test for two-sample variance (between the expected values, i.e., the summed effect of individual treatments, and the observed values, i.e., the effect in combination treatment, subtracting values from vehicle control in all cases). This was followed with a two-tailed t-test assuming equal or unequal variances, based on the obtained *p*-value in the previous test. Venn diagrams were prepared using Venny software [43], while gene ontology figures were generated using ShinyGo 0.85.1 software [44]. All figures were assembled and edited in Adobe Illustrator 2025 from Adobe Inc. (San Jose, CA, USA).

## 3. Results

### 3.1. Analysis of FGFR and JAK/STAT Pathways in AMLs from Public Databases

**Figure 1:**
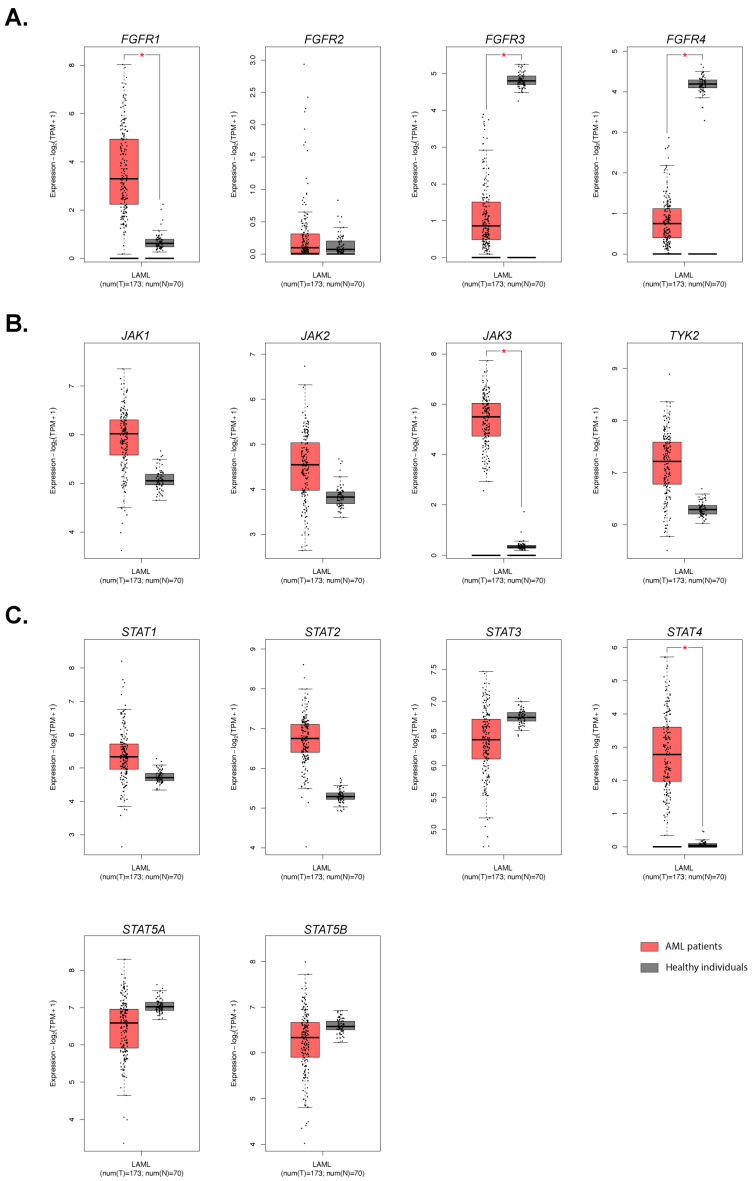
Comparison of selected gene expressions in healthy individuals vs. AML patients. Using GEPIA2.0 software, data from 173 AML patients were compared to data from 70 healthy individuals to study differences in the expression of *FGFR1-4* (**A**), *JAK1-3* and *TYK2* (**B**), and *STAT1-4* and *STAT5A-B* (**C**). The data are presented as Log_2_FoldChange > 2. The statistical significance is presented as * (*p* < 0.01).

We began our investigation with the analysis of the publicly available databases with genomic and transcriptomic data of AML patients and healthy individuals. The genes selected for this analysis were connected to FGFR and JAK/STAT signal transduction pathways (*FGFR1*, *FGFR2*, *FGFR3*, *FGFR4*, *JAK1*, *JAK2*, *JAK3*, *TYK2*, *STAT1*, *STAT2*, *STAT3*, *STAT4*, *STAT5A*, and *STAT5B*). In the first analysis, data from cBioPortal, which contains data from 6709 individuals, was used to search if the mutations in the above-listed genes occur in AML blasts [45,46]. Most of the studied genes were rarely found to be mutated (<1% of cases), except for *JAK2*, where mutation V617F was found in 229 cases, contributing to 6% of all AML patients in this study (Supplementary Figure S1). This mutation causes the JAK2 protein to be constitutively active, which is very common in certain hematological diseases [47,48]. In the second analysis, the expression profiles of the aforementioned genes were studied. For this purpose, Gene Expression Profiling Interactive Analysis 2 (GEPIA2) software was used to access The Cancer Genome Atlas (TCGA), where RNAseq data from 173 AML patients (from TCGA database) and 70 healthy individuals (from The Genotype-Tissue Expression-GTEx project’s database) were available [49,50,51]. With Log_2_FoldChange of 2 and *p*-value of 0.01 cutoffs, we identified the changes in the expression of the genes in patients, as compared to healthy individuals. As presented in Figure 1, the expressions of three genes, *FGFR1*, *JAK3,* and *STAT4*, were significantly higher, while the expressions of *FGFR3* and *FGFR4* were significantly lower in AML patients than in healthy people. These results indicate that disturbances in FGFR and JAK/STAT pathways are quite common in AML and are worthy of further investigation.

### 3.2. Differentiation and Apoptosis in AML Cells in Response to PRI5202 and Fludarabine

1,25D and its analogs at concentrations that induce differentiation are not toxic to AML cells, and their anticancer effect is due to differentiation and loss of immortalization [52]. However, Fludarabine, which is an anticancer drug, may be toxic to proliferating cells. In our experiments, we wanted to use Fludarabine at sub-toxic concentrations because we were interested in its role in gene transcription and protein translation. In that part of our investigation we used the U937 cell line, which is a pro-monocytic, human myeloid leukemia cell line that was isolated from the histiocytic lymphoma of a 37-year-old male and was used in the past as the experimental model of monocyte and macrophage differentiation [53]. Because from our previous work we had known that U937 cells overexpressing FGFRs are more sensitive to 1,25D than the wild-type or mock-transfected cells, we also used U937-Empty (mock-transfected), U937-FGFR1 (FGFR1-transfected), and U937-FGFR2 (FGFR2-transfected) cells [20]. We refer to the untransfected U937 cells as wt-U937 in this paper. At first, the inhibitory concentration (IC)_50_ of Fludarabine towards wt-U937 and U937-Empty cells was established, and the results are presented in Supplementary Materials (Figure S2). The U937-Empty cells were more sensitive to Fludarabine-induced toxicity than wt-U937 cells, with a Fludarabine IC_50_ of 315 nM. Therefore, we decided to use Fludarabine at 315 nM concentration in all further analyses using all studied cells. We also used our best 1,25D analog, PRI5202 at 10 nM concentration, which was established in our earlier works.

**Figure 2:**
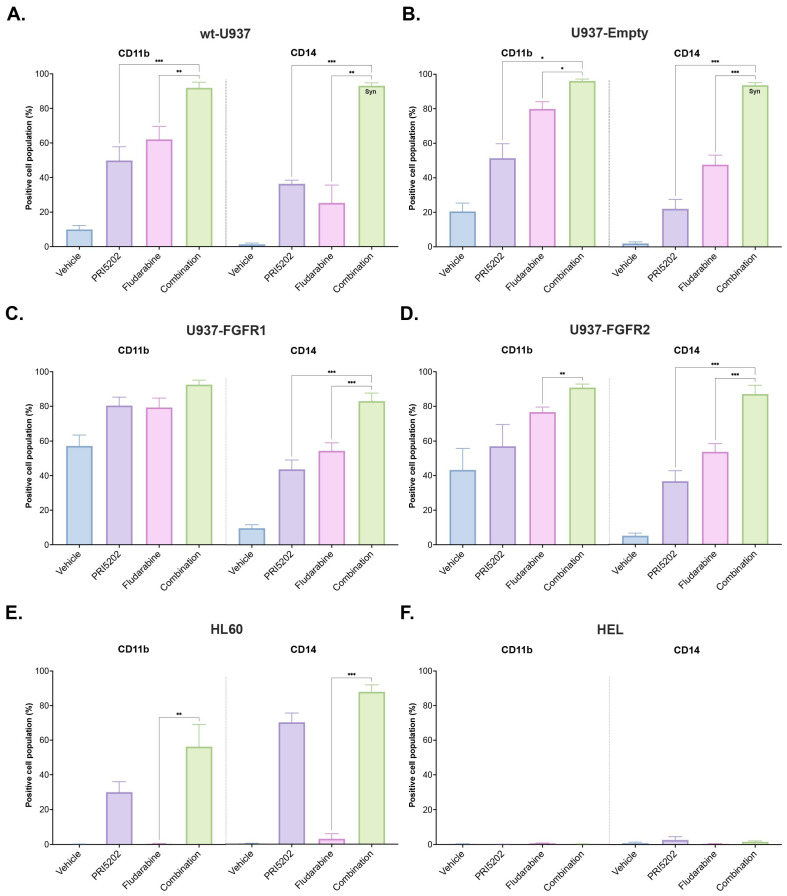
CD11b and CD14 proteins on AML cell lines exposed to PRI5202 or/and Fludarabine. CD11b and CD14 cell surface proteins were detected using flow cytometry in wt-U937 (**A**), U937-Empty (**B**), U937-FGFR1 (**C**), U937-FGFR2 (**D**), HL60 (**E**), and HEL (**F**) cells exposed to vehicle or to 10 nM PRI5202 or/and 315 nM Fludarabine for 96 h, stained with fluorophore-conjugated antibodies, and analyzed on Becton Dickinson Accuri C6 flow cytometer. The data are represented as mean ± SEM from at least 3 replicates, with statistical analyses of combination treatment vs. individual treatments. Whenever the synergistic effect was found, it was marked as Syn on the respective graph. Significant differences are represented as * (*p* < 0.033), ** (*p* < 0.002), or *** (*p* < 0.001).

In our next experiments, the levels of monocytic cell surface markers were studied in flow cytometry. It has been established before that differentiation of AML cells in response to 1,25D or to its analogs is a slow process, which needs gene transcription and protein translation, and reaches a plateau between 72 and 96 h from exposure to the drug [54]. Thus, we decided to study differentiation in response to 10 nM PRI5202 or/and 315 nM Fludarabine after 96 h. As shown in Figure 2A–D, untreated wt-U937 and untreated U937-Empty cells have low levels of CD11b and CD14 antigens on the cell surface. Untreated U937-FGFR1 and U937-FGFR2 cells have constitutively high levels of CD11b antigen but low CD14 levels on their surface. Exposure to PRI5202 or Fludarabine increases the levels of CD14 in all cell sublines, while CD11b is increased only in wt-U937 and U937-Empty cells. We were interested in whether there was any cooperativity in actions of PRI5202 and Fludarabine. Synergy was calculated according to the Response Additivity Model [55] and was observed in the upregulation of the CD14 cell surface marker in wt-U937 and U937-Empty cells. In U937-FGFR2 cells, both antigens were upregulated in an additive manner.

We were also interested whether exposure of the cells to PRI5202 or/and Fludarabine could trigger apoptosis in the above cell lines. As shown in Supplementary Figure S3A–D, the viability of wt-U937 cells was not affected by any of the compounds, while the viable cell populations in all plasmid-transfected cells were affected by Fludarabine alone, and progressed to the late apoptotic phase. PRI5202 alone did not contribute to such changes.

In the next step, the responses of other cell lines originating from myeloproliferative diseases to the proposed treatment were studied. HL60 cells originate from a 36-year-old woman who suffered from AML M2, which is now referred to as AML with maturation [56,57]. These cells carry many mutations, such as *MYC* amplification [58], deletion of part of *TP53* [59], or *NRAS* mutations [60]. These cells respond well with differentiation to 1,25D or to vitamin D analogs [57]. HEL cell line originated from a 30-year-old male suffering from AML M6, which was a complication after treatment against Hodgkin lymphoma [61]. HEL cells were shown as unresponsive to 1,25D or to vitamin D analog [62]. As seen in Figure 2E,F, PRI5202 alone enhanced the levels of both CD11b and CD14 proteins on HL60 cells, while Fludarabine did not affect them. HEL cells did not respond with differentiation to either of the treatments. Fludarabine induced some early apoptosis in HEL cells (Supplementary Figure S3E,F).

### 3.3. RNAseq Analysis of PRI5202 and Fludarabine in AML Cell Lines

Having established the concentrations of PRI5202 and Fludarabine, which together induce stronger differentiation than each of the compounds alone, we decided to investigate the changes in the global transcriptome in response to the compounds alone or to their combination and compare them to the control cells (exposed to the vehicle). Since we were interested not only in transcription of the early primary target genes but also in the secondary ones, we looked at transcriptomes after 48 h of exposure to the tested compounds. The cell lines investigated were wt-U937, U937-Empty, U937-FGFR1, U937-FGFR2, HL60, and HEL. Since samples analyzed in this assay were not in replicates, the data from RNAseq experiment were considered as indicative to give direction to further experiments.

**Figure 3:**
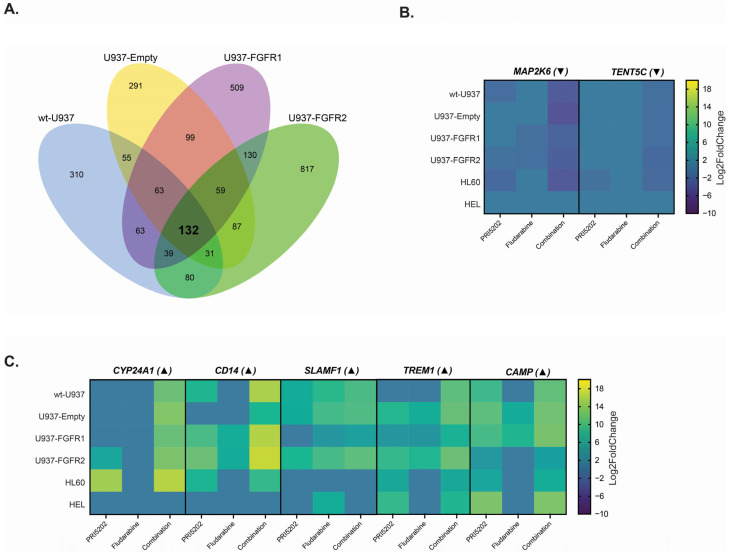
Global transcriptomes in AML cell lines exposed to PRI5202 or/and Fludarabine. Wt-U937, U937-Empty, U937-FGFR1, U937-FGFR2, HL60, and HEL cells were exposed to vehicle or to 10 nM PRI5202 or/and 315 nM Fludarabine for 48 h, and transcriptomic sequencing was performed. The initial DEG data were analyzed using NovoMagic software. Protein-coding DEGs were identified with |Log_2_FoldChange| of 1 and *p*-adj < 0.05. The DEGs whose expression levels enhanced synergistically after exposure to combination treatment were identified using Formula (1). The Venn diagram of the genes regulated in a synergistic mode in all U937 cell sublines is presented (**A**). The genes from this group that were most strongly downregulated (▼; **B**) or upregulated (▲; **C**) are presented as heatmaps.

Using the NovoMagic software from Novogene, DEG data from all six cell lines were retrieved for the samples exposed to 10 nM PRI5202 or/and 315 nM Fludarabine vs. vehicle, with a cutoff of |Log_2_FoldChange| of 1 and adjusted *p*-value < 0.05. Protein-coding genes were filtered, and using Power Query tool from Microsoft, commonly up- and downregulated genes were identified. Using Formula (1) given in the Materials and Methods section, genes were categorized as being regulated in a synergistic manner or not. To rank the genes, a “synergy score” was assigned to each gene, based on Formula (2) in the Materials and Methods section. Then, the synergistically regulated genes were analyzed to find those that were common for all cell lines, but there were few of them. There were 132 common genes found in all U937 sublines (Figure 3A), and we decided to analyze them in more detail. Out of this group, the most strongly upregulated genes were selected for further investigation (Figure 3C). Genes encoding Cytochrome P450 1,25D hydroxylase (*CYP24A1*), CD14 (*CD14*), CD354 (*TREM1*), and cathelicidin antimicrobial peptide (*CAMP1*) were synergistically upregulated not only in all U937 cell sublines but also in HL60 cells. It is noteworthy that *CAMP* gene was upregulated synergistically by a combination treatment also in HEL cells, which are almost unresponsive to PRI5202 (Figure 3C). Further, using iDEP2.0 software [63] and Gene Ontology (GO) enrichment [64], the genes synergistically upregulated in all U937 sublines were analyzed for their biological significance. Using k-means clustering algorithm with the genes normalized by the standard deviation (SD) of their expression, the genes were ranked into four clusters. Cluster 2 included the genes with the highest synergy score, such as *CAMP*, *CD14*, *CYP24A1*, *TREM1*, and *SLAMF1* (Supplementary Figure S4A). GO analysis of cluster 2 revealed that defense and inflammatory responses were synergistically upregulated by PRI5202 and Fludarabine in all U937 cell sublines. The top 10 enriched pathways, shown in Supplementary Figure S4B, revealed that inflammation was the most strongly upregulated process, while response to external stimuli and immune processes were composed of the highest number of genes. The DEG analysis also revealed genes which were regulated in an antagonistic manner by a combination of 10 nM PRI5202 and 315 nM Fludarabine. The numbers of genes regulated in a synergistic and antagonistic manner are given in Supplementary Table S3.

### 3.4. Further Investigation of Synergy Between PRI5202 and Fludarabine in AML Cell Lines

At the next step, we decided to investigate the expressions of the genes for which the synergistic upregulation in response to 10 nM PRI5202 and 315 nM Fludarabine was strong in RNAseq experiments, using RT-qPCR, which is quantitative and more reproducible than the previous method. We selected five genes, which were the most strongly upregulated in a synergistic manner in wt-U937 cells. These five genes were also strongly and synergistically upregulated in the remaining U937 sublines, and four of them were synergistically upregulated in HL60 cells. Since only one of these genes was synergistically upregulated in HEL cells, we decided to discontinue the experiments using this cell line (see Table S4).

The following genes were selected:

*CYP24A1* encoding Cytochrome P450, a 1,25D hydroxylase enzyme found in the inner membrane of mitochondria, which degrades 1,25D into the calcitroic acid [65,66].

*CD14* encoding CD14, which is found on the cell surface or as a secreted molecule. The protein is found on the surface of dendritic cells, natural killers, and macrophages. CD14 recognizes pathogen-associated molecular patterns, as well as damage-associated molecular patterns. CD14 participates in macrophage activation, thereby activating the innate immune system [67,68,69].

*SLAMF1* (Signaling Lymphocytic Activation Molecule Family Member 1) encoding CD150, which supports functions of various activated immune cells, such as T helper cells, natural killers, dendritic cells, and macrophages, but also facilitates infection by measles virus, leading to immunosuppression [70].

*TREM1* (Triggering Receptor Expressed On Myeloid Cells 1) encoding CD354, which is present on the surface of neutrophils and macrophages and is associated with their anti-fungal roles by strengthening innate immune responses [71,72].

*CAMP* encoding cathelicidin antimicrobial peptide, the 18 kDa precursor of human cationic antimicrobial protein LL-37, which disrupts the membranes of pathogens. LL-37 is secreted in vesicles from immune cells such as natural killer cells and macrophages, acting as an antimicrobial protective agent and as a chemo-attractant to T cells [73,74].

In addition, *ITGAM* encoding CD11b was selected for RT-qPCR analysis. This gene encodes a subunit alpha of heterodimer integrin, expressed in various immune cells, including monocytes and macrophages. The protein dimer plays a role in intracellular adhesion, activation, and motility of leukocytes during inflammation and phagocytosis and is very often used as a marker of the myeloid pathway of differentiation [75]. In RNAseq, this gene was synergistically upregulated in all U937 sublines, as well as in HL60 cells.

**Figure 4:**
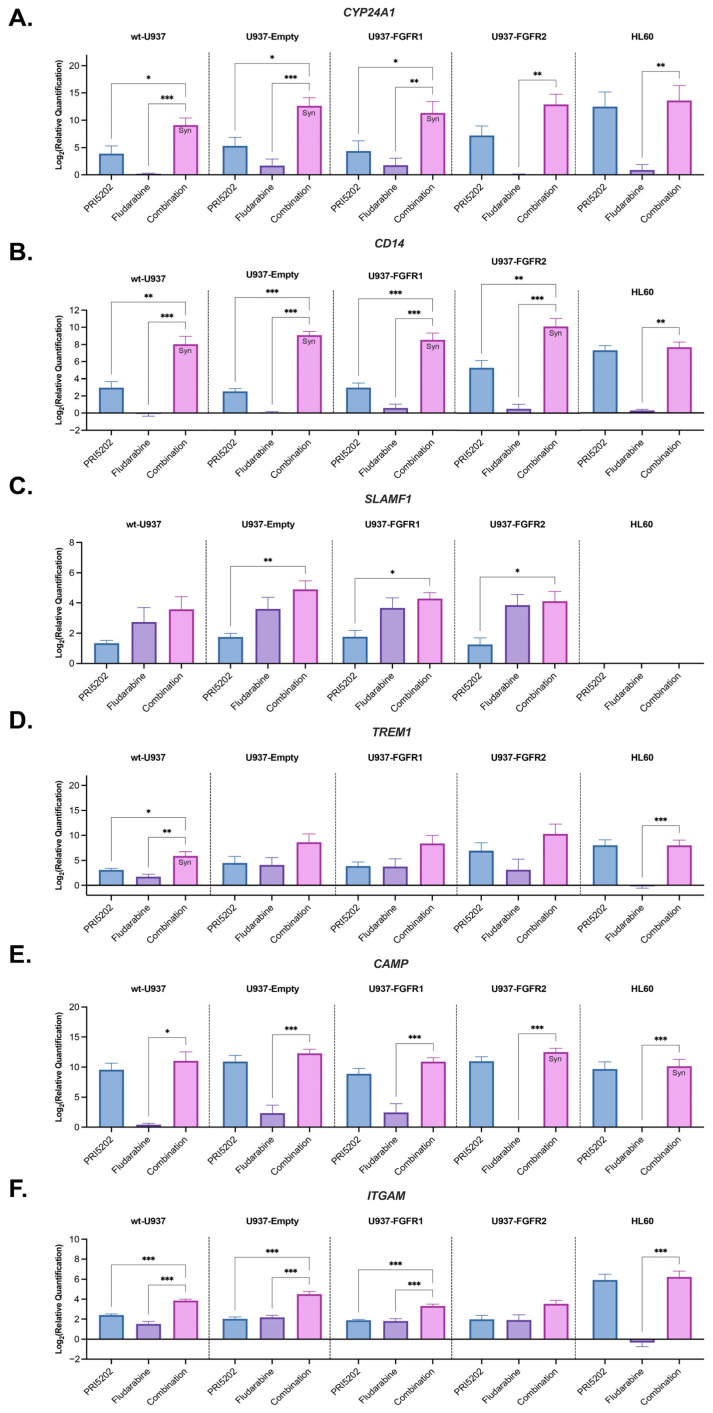
Transcript levels in AML cell lines in response to PRI5202 or/and Fludarabine. Total RNA was isolated from wt-U937, U937-Empty, U937-FGFR1, U937-FGFR2, and HL60 cells, exposed to vehicle or to 10 nM PRI5202 or/and 315 nM Fludarabine for 48 h, and reverse-transcribed into cDNA. The expression levels of *CYP24A1* (**A**), *CD14* (**B**), *SLAMF1* (**C**), *TREM1* (**D**), *CAMP* (**E**), and *ITGAM* (**F**) were normalized to *GAPDH*. The normalized expression levels of these genes in the PRI5202, Fludarabine, and combination treatment samples were quantified relative to the vehicle control samples using the 2^−ΔΔCt^ method. The data are presented as mean ± SEM from 4 biological replicates, with statistical analyses of combination treatment vs. individual treatments. Whenever the synergistic effect was found, it was marked as Syn on the respective graph. Significant differences are represented as * (*p* < 0.033), ** (*p* < 0.002), or *** (*p* < 0.001).

As presented in Figure 4, expressions of almost all studied genes were upregulated after exposure to 10 nM PRI5202 and a combination of PRI5202 and 315 nM Fludarabine. Not all experiments revealed synergy between PRI5202 and Fludarabine; however, some of them did. The synergy was observed in the expression of *CYP24A1* in wt-U937, U937-Empty, and U937-FGFR1. For *CD14*, the synergy was observed in all sublines of U937 cells, for *TREM1* only in wt-U937, and for *CAMP* in U937-FGFR2 and HL60 cells.

**Figure 5:**
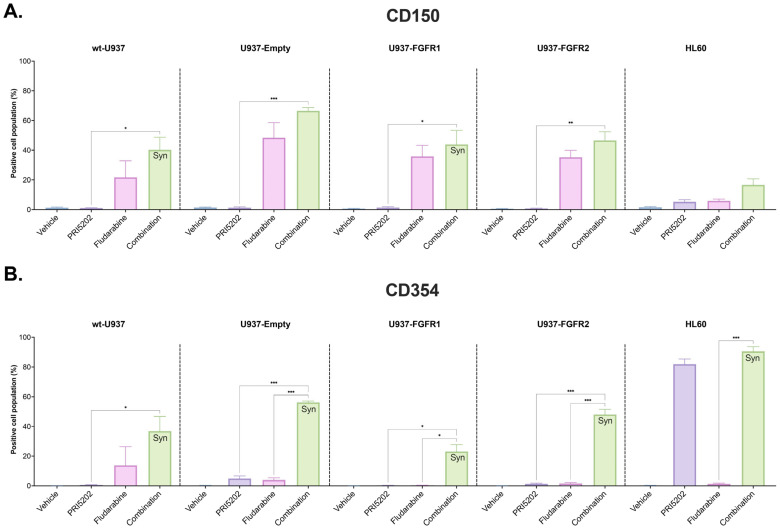
CD150 and CD354 proteins on AML cell lines exposed to PRI5202 or/and Fludarabine. Protein levels of the surface markers CD150 (**A**) and CD354 (**B**) were assessed by flow cytometry in wt-U937, U937-Empty, U937-FGFR1, U937-FGFR2, and HL60 cells, exposed to vehicle or to 10 nM PRI5202 or/and 315 nM Fludarabine for 96 h. The data are represented as mean ± SEM from 4 biological replicates, with statistical analyses of combination treatment vs. individual treatments. Whenever the synergistic effect was found, it was marked as Syn on the respective graph. Significant differences are represented as * (*p* < 0.033), ** (*p* < 0.002), or *** (*p* < 0.001).

We also wanted to verify if upregulation of the gene expression was followed by a respective protein translation. For that investigation, we selected CD150 and CD354, which are cell surface markers. The graphs in Figure 5 show the levels of CD150 and CD354 surface markers on U937 cell sublines and on HL60 cells. There was a significant synergistic effect observed in the level of CD354, encoded by *TREM1*, in all sublines of U937 and in HL60 cells. It should be noted that neither PRI5202 nor Fludarabine individually increased the level of this receptor, while in combination they did. Again, it is noteworthy that Fludarabine alone increased levels of CD150 in all U937 cells.

### 3.5. Investigation on the Mechanism of Action of Fludarabine

As mentioned in the introduction, Fludarabine was reported to inhibit the activation of STAT1 [34,35]. We also tested if that was the case with our experimental models. To investigate that, U937 cells from all sublines and HL60 cells were pre-exposed to 315 nM Fludarabine for 24 h or to the vehicle control, and then exposed to 100 ng/mL IFNγ for 15 min. The cell lysates were analyzed in Western Blots using the antibodies against phospho-STAT1 and STAT1. In our experiments, neither the inhibition of STAT1 phosphorylation nor a decrease in total STAT1 was observed.

**Figure 6:**
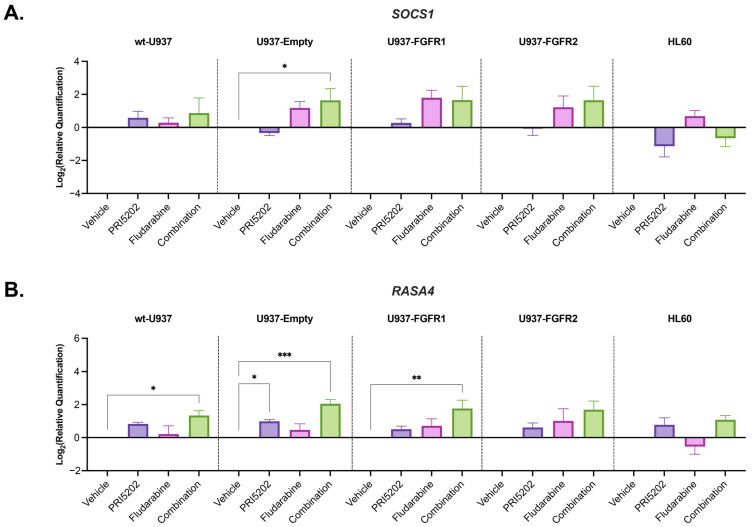
The investigation of the STAT pathway in AML cell lines exposed to PRI5202 or/and Fludarabine. *SOCS1* (**A**) and *RASA4* (**B**) gene expression, normalized to *GAPDH* expression within samples, was tested using the RT-qPCR method in all U937 sublines and HL60 cells exposed to vehicle or 10 nM PRI5202 or/and 315 nM Fludarabine for 48 h. The data are represented as mean ± SEM from 4 biological replicates, with statistical analyses of Treatment Vs. Control samples. Significant differences are represented as * (*p* < 0.033), ** (*p* < 0.002) or *** (*p* < 0.001).

Therefore, we analyzed the RNAseq data again and found out that *SOCS1* and *RASA4* gene expressions were slightly upregulated after exposure of the cells to Fludarabine, and even more after exposure to combination of PRI5202 and Fludarabine (Supplementary Figure S4C). *SOCS1* encodes the most potent member of SOCS (Suppressors of Cytokine Signaling) protein family, a known feedback inhibitor of the JAK/STAT pathway, upregulated in response to IFNγ. This protein has dual activity. It has a kinase inhibitory region, which directly interacts with and blocks the JAK’s kinase, but also acts as an ubiquitin ligase leading to degradation of JAK2 [76]. *RASA4* encodes Ras p21 protein activator 4, which belongs to the guanine nucleotide exchange factors (GEF) superfamily. RASA4 translocates to the plasma membrane upon intracellular Ca^2+^ buildup. Once activated, it suppresses the Ras/MAPK-modulated cell growth and differentiation. This protein is also known as Calcium-Promoted Ras Inactivator (CAPRI). RASA4 is expressed in healthy blood cells, while in most cases of Juvenile Myelomonocytic Leukemia, the transcript was found to be epigenetically silenced [77]. RASA4 protein is essential for chemotactic migration of neutrophils, suggesting its importance for the innate immune system [78,79]. We hence decided to investigate in RT-qPCR the expression of these two genes with cells exposed to vehicle, or to 10 nM PRI5202 or/and 315 nM Fludarabine for 48 h (Figure 6). In these experiments the upregulation up to 4-times of *SOCS1* expression in comparison to vehicle-treated samples was observed upon Fludarabine exposure (not statistically significant) (Figure 6A), and up to 4-times of *RASA4* expression was observed upon PRI5202 exposure when compared to vehicle (Figure 6B).

**Figure 7:**
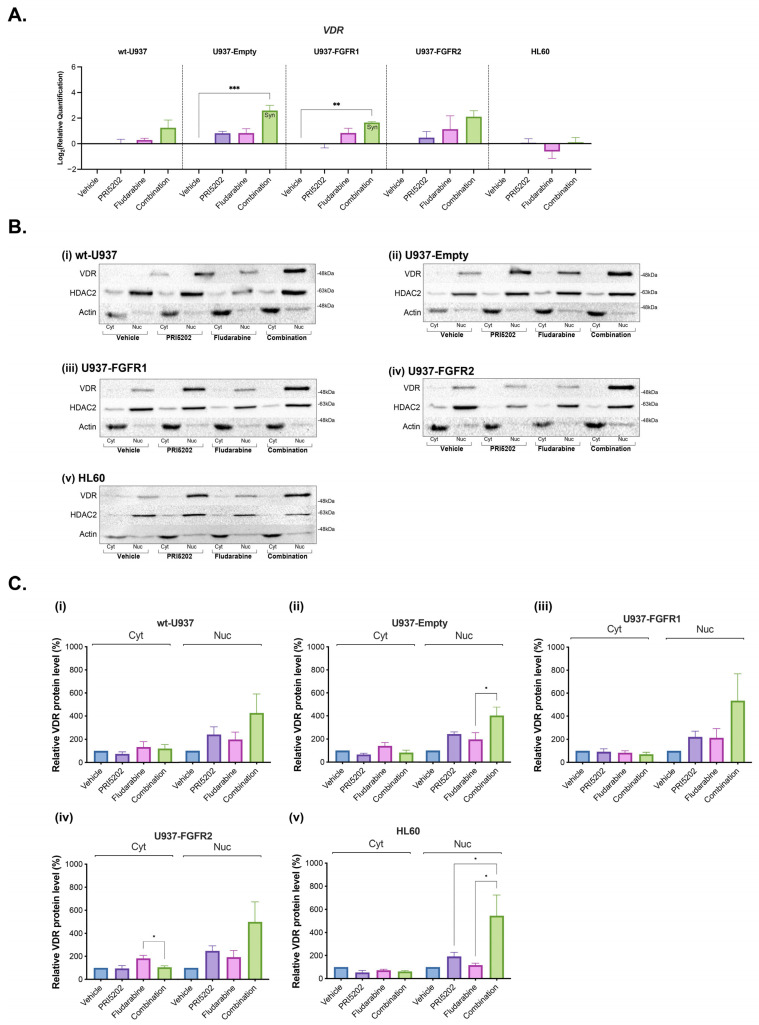
The investigation of VDR gene and protein in AML cell lines exposed to PRI5202 or/and Fludarabine. *VDR* gene expression normalized to *GAPDH* expression within samples was tested using the RT-qPCR method in all U937 sublines and HL60 cells exposed to vehicle or 10 nM PRI5202 or/and 315 nM Fludarabine for 48 h (**A**). The data are represented as mean ± SEM from 4 biological replicates, with statistical analyses of treatment vs. control samples. VDR protein levels in all U937 sublines and HL60 cells exposed to vehicle or 10 nM PRI5202 or/and 315 nM Fludarabine for 24 h were probed in Western blotting (**B**) in wt-U937 (i), U937-Empty (ii), U937-FGFR1 (iii), U937-FGFR2 (iv) and HL60 (v) cells. The samples were lysed into cytoplasmic (Cyt) and nuclear (Nuc) fractions. HDAC2 (for nuclear fractions) and actin (for cytoplasmic fractions) were used as controls of equal protein loading. The quantifications of blots from 4 biological replicates of wt-U937 (i), U937-Empty (ii), U937-FGFR1 (iii), U937-FGFR2 (iv) and HL60 (v) cells are presented (**C**). Whenever the synergistic effect was found, it was marked as Syn on the respective graph. Significant differences are represented as * (*p* < 0.033), ** (*p* < 0.002), or *** (*p* < 0.001).

In order to understand if the effects of the drugs are mediated Via VDR, we decided to study *VDR* gene expression and VDR protein levels in all U937 sublines and in HL60 cells exposed to 10 nM PRI5202 or/and 315 nM Fludarabine for 48 h. As presented in Figure 7A, PRI5202 alone did not upregulate the expression of *VDR*, while in combination with Fludarabine, it did in U937-Empty and U937-FGFR1 sublines. However, it has been known that the amount of VDR in the cell is regulated by its stability rather than by its gene expression. VDR protein is unstable when not bound to its ligand, but upon ligation, it becomes stable and translocates to the cell nucleus. These effects of VDR stabilization and nuclear translocation are fast and can be seen after 1 h [80,81], but start to decrease after 48 h (our unpublished observation). Thus, we have performed Western blotting experiments to investigate the level and the cellular location of VDR at 24 h after treatments. According to our earlier experiences, histone deacetylase 2 (HDAC2) was chosen as a control of equal protein loading for nuclear fractions and actin for cytosolic fractions [82]. Representative blots, which are shown in Figure 7B indicate that the combination of PRI5202 and Fludarabine caused accumulation of VDR in the cell nuclei to the extent higher than either PRI5202 or Fludarabine alone. The densitometry of blots from four biological replicates is presented in Figure 7C.

### 3.6. Effects of PRI5202 and Fludarabine in Bone Marrow Cells from Healthy Donors and in Bone Marrow Blasts from Patients with Hematological Malignancies

Finally, we wanted to verify if 10 nM PRI5202 or/and 315 nM Fludarabine had similar effects towards blasts from patients with hematological malignancies as it had to AML cell lines. We received bone marrow aspirates from 17 donors from the Department of Hematology, Blood Neoplasms, and Bone Marrow Transplantation from Wrocław Medical University, after obtaining informed consent from the donors. The donors were either healthy (5 donors), or suffered from MDS (7 donors), AML (4 donors), or chronic myelomonocytic leukemia (CMML; 1 person). The characteristics of the donors whose bone marrows were tested in this study are presented in Supplementary Table S2. Bone marrow mononuclear cells, which in patients with malignancies were mostly blasts, were then exposed to vehicle or to 10 nM PRI5202 or/and 315 nM Fludarabine for 96 h, and afterwards the levels of CD11b (n = 17), CD14 (n = 17), CD150 (n = 5), and CD354 (n = 5) cell surface markers were examined in flow cytometry (please note: one sample was treated with vehicle and PRI5202 only—before the concentration of Fludarabine was established; and CD150 and CD354 as a target of study was identified later; therefore, fewer samples were tested for these two proteins). The mononuclear cells were also stained with 7AAD to distinguish viable from dead cells. The dead cells were outgated from further analyses; thus, the levels of cell surface markers were measured only in viable cells. An example of the gating procedure is presented in Supplementary Figure S5.

**Figure 8:**
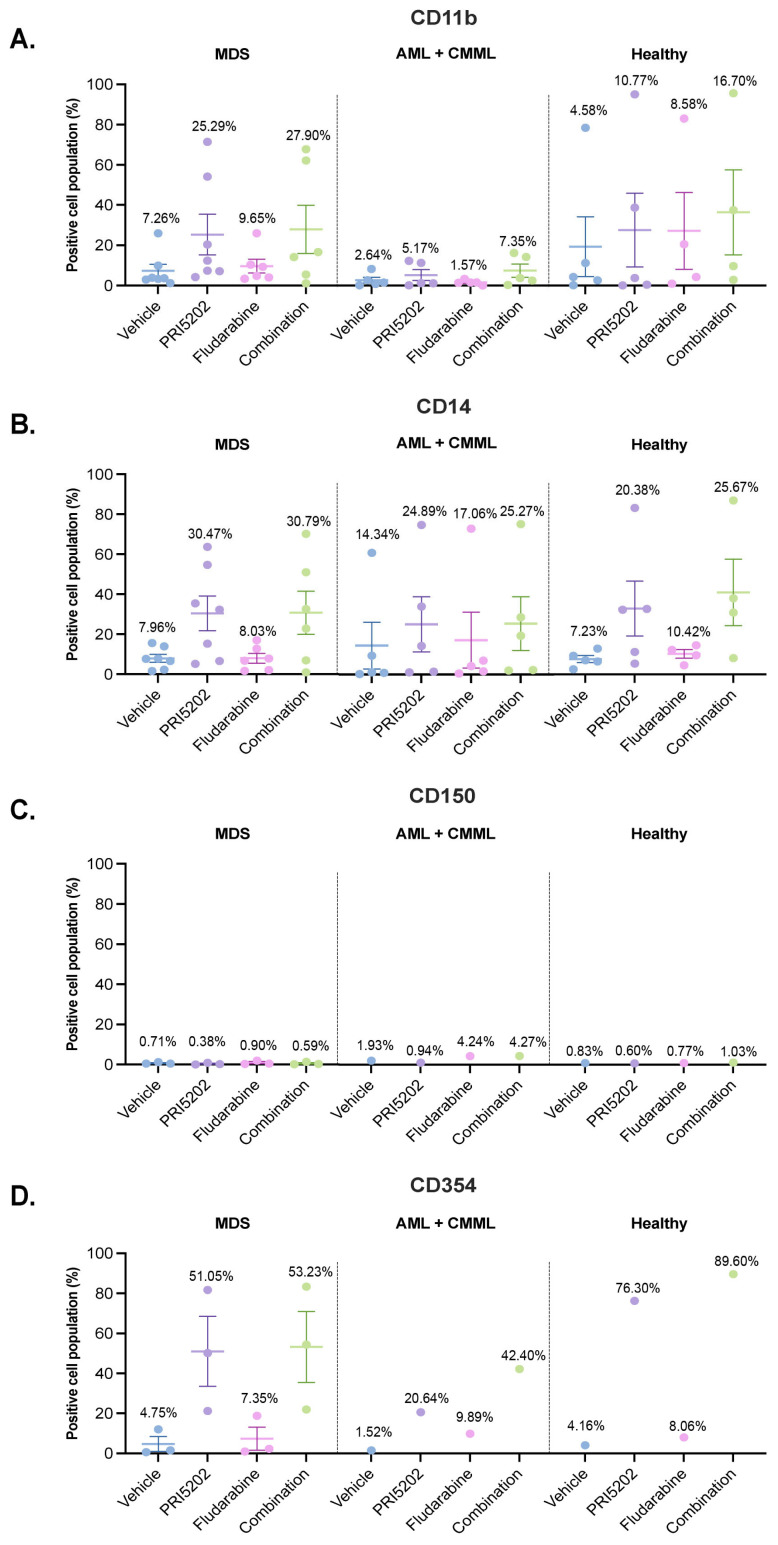
Differentiation of bone marrow mononuclear cells exposed to PRI5202 or/and Fludarabine. The levels of CD11b (**A**), CD14 (**B**), CD150 (**C**), and CD345 (**D**) on bone marrow mononuclear cells obtained from human donors, after 96 h exposure to vehicle, 10 nM PRI5202, or/and 315 nM Fludarabine, were measured in flow cytometry. The percentages of cells positive for the given cell surface marker are presented. The samples are categorized as MDS, AML + CMML, or Healthy, according to the donor’s diagnosis. Each dot represents the value obtained for a given sample, the dash represents the mean value, and the whiskers represent SEM for the group. The mean values are presented above each graph.

As presented in Figure 8, the highest differentiation in response to PRI5202 was observed in the blasts from patients suffering from MDS. Unfortunately, the number of donors in each group was too small to perform statistical analysis; therefore, we do not know if there was any synergy between 10 nM PRI5202 and 315 nM Fludarabine; however, the mean values of surface marker-positive cells were in some groups higher in combination than in individual treatments.

**Figure 9:**
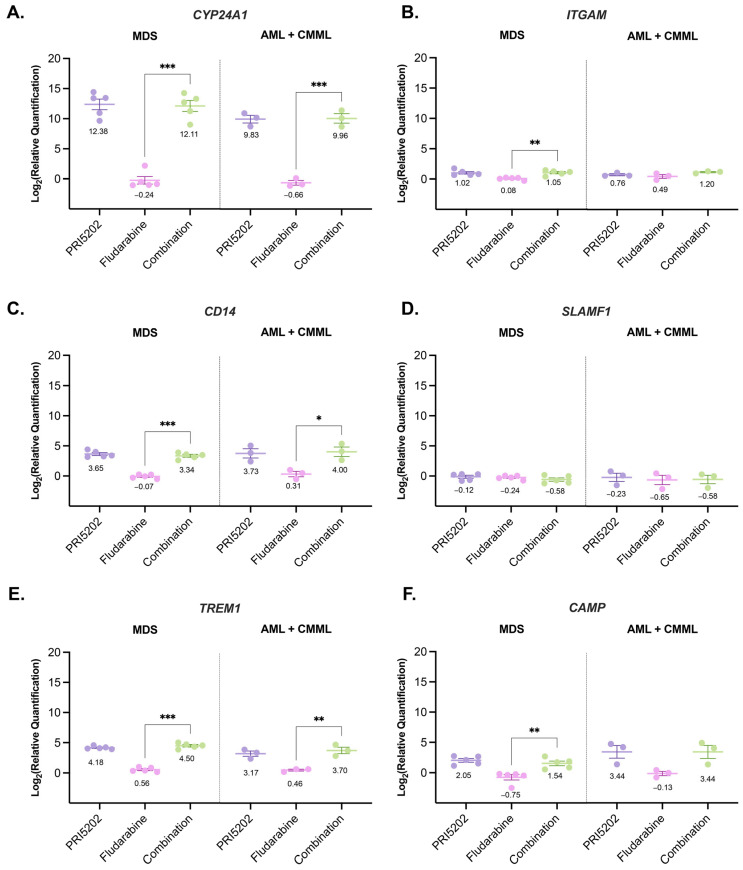
Gene expression in human bone marrow mononuclear cells exposed to PRI5202 or/and Fludarabine. RT-qPCR experiments were performed on bone marrow mononuclear cells isolated from human donors and treated (48 h) (n = 8) with vehicle, 10 nM PRI5202, or/and 315 nM Fludarabine to check the transcript levels of *CYP24A1* (**A**), *ITGAM* (**B**), *CD14* (**C**), *SLAMF1* (**D**), *TREM1* (**E**), and *CAMP1* (**F**). The expression levels were normalized with *GAPDH* expression in the sample, and cross-treatment effects were quantified to the normalized expression in vehicle treated sample, using the 2^−ΔΔCt^ method. The data is presented for MDS (n = 5) and AML + CMML (n = 3) grouped donors. Each dot represents the value obtained for a given subject, the dash indicates the mean, and the whiskers SEM for the group. Significant differences in subject group means are represented as * (*p* < 0.033), ** (*p* < 0.002), or *** (*p* < 0.001). The mean values are presented below each graph.

We were also interested if the proposed treatment influenced the mRNA levels of these genes, which were identified in RNAseq as synergistically regulated by PRI5202 and Fludarabine. Unfortunately, in the samples from healthy donors, the number of cells was not sufficient for RNA isolation and reverse transcription. Moreover, due to inadequate quality and/or quantity, four further samples were rejected from the RT-qPCR examination (2 MDS and 2 AML). Therefore, the blasts from 8 patients (5 MDS, 2 AML, and 1 CMML) were exposed to vehicle, 10 nM PRI5202, or/and 315 nM Fludarabine for 48 h, and expressions of *CYP24A1*, *ITGAM*, *CD14*, *SLAMF1*, *TREM1*, and *CAMP* were measured in RT-qPCR. The said gene expressions were normalized to *GAPDH* and quantified to the vehicle treated sample (Figure 9), as described in Materials and Methods.

## 4. Discussion

AML can affect people of all ages, but it is diagnosed predominantly in elderly patients. Five-year survival is strongly dependent on age, ranging from 62% in patients younger than 50 to 9.4% in patients older than 65. The difference is mostly due to the fact that older patients are not fit for the standard intensive chemotherapy that includes cytarabine (AraC) and anthracycline. Therefore, alternative low-intensive regimens with palliative intent are used for older and less fit patients. The efficacies of these regimens have been improving recently [1,83], but there is still a need for progress in this area.

The goal of this study was to test the combination of cell-differentiating agent, with a low concentration of Fludarabine, in in vitro and ex vivo models of AML and MDS. The cell-differentiating agent, PRI5202, is the analog of an active metabolite of vitamin D, which is more active than 1,25D in inducing cell differentiation, but less calcemic [36]. Fludarabine, when used at low concentrations, is thought to inhibit the activity of the STAT1 transcription factor [34], which is often upregulated in AML blasts [26]. Our analysis of the publicly available databases indicated that mutation *JAK2* V617F was found in 6% of blasts from AML patients, and that expressions of *FGFR1* and *JAK3* were significantly higher in AML patients than in healthy people (Figure S1 and Figure 1). Since overactivation of JAK2, JAK3, or FGFR1 leads to overactivation of STAT1, we decided to test Fludarabine as a potential inhibitor of these signal transduction pathways.

Both drugs used in our study were applied at concentrations which did not induce immediate apoptosis or necrosis in the cells (Figure S2). In contrast, we observed differentiation towards monocytes in the majority of the cell lines and in blasts from the patients (Figure 2 and Figure 8). Moreover, in all U937 sublines and HL60 cells, we observed a synergy between PRI5202 and Fludarabine in a pro-differentiation activity (Figure 2 and Figure 5).

The mode of action of PRI5202 is similar to that of 1,25D. These agents bind to the ligand binding domain of nuclear VDR with similar affinity [36]. Upon ligation, VDR binds to the vitamin D response elements (VDRE) in promoter regions of the target genes and activates their transcription. Many of these genes regulate various aspects of the immune system [22,23]. Some of them are immediate targets of VDR, while the others are regulated as secondary targets. In our experiments, we wanted to observe both types; thus, we decided to investigate RNAseq after relatively long (48 h) exposure time. As presented in Figure 3, the cells responded to combination of PRI5202 and Fludarabine in upregulation of many genes involved in body defense and inflammation. RT-qPCR confirmed that the genes encoding proteins important for functions of macrophages and neutrophils were upregulated (Figure 4), and flow cytometry experiments of CD150 and CD354 revealed that gene upregulation was followed by protein translation (Figure 5).

In our experiments, we were unable to observe the inhibition of STAT1 phosphorylation by Fludarabine, which was earlier reported in other cell models [34,84]. However, the RNAseq data revealed that Fludarabine at low concentration is a potent regulator of gene transcription (Figure 3 and Figure S7). There were numerous genes whose transcription was regulated by 315 nM Fludarabine in the cell lines tested (e.g., Figure S7B). As an example, 315 nM Fludarabine alone upregulated expression of *SLAMF1*, which was followed by the translation of the encoded protein (Figure 4 and Figure 5).

Since Fludarabine is a purine analog, we expect that it may exert epigenetic activity [85,86]; however, the exact mechanism of regulation of transcription by Fludarabine needs to be studied. An interesting example of a well-known antimetabolite with a secondary epigenetic role is AraC, a synthetic pyrimidine nucleoside, converted intracellularly to active AraC-triphosphate (AraCTP), which alters the activity of DNA polymerases [87]. AraC does not completely block elongation and remains embedded in DNA, but it is not methylated as efficiently in vivo by DNA methylase as cytosine, thus altering the global methylation pattern in dividing cells [88]. That exact mechanism of action cannot be shared by Fludarabine, which is not a cytosine analog; however, the epigenetic effects similar to these of thiopurine [89] or cladribine [90] may be considered.

Another issue that needs attention is which subtypes of AML respond well to the proposed combination treatment. It has been documented in the past that patients whose blasts harbor *IDH*-R132H mutation respond well to 1,25D due to the higher expression of *VDR* [21]. In our cell line models, we observed that wt-U937 cells responded to combination of PRI5202 and Fludarabine with higher numbers of regulated genes than HL60 and HEL cells. U937 cells harbor the activating mutation *JAK3*-M511I which is present in about 3–10% of AML cases [91]. U937 cells with overexpression of FGFRs responded with higher numbers of regulated genes than wt-U937 cells (Figure 3). Mutations in *FGFR*s are not common among AML patients, but overexpression is much more frequent (Figure 1) [92] and often leads to the overactivation of JAK/STAT pathways [93]. We hypothesize that AML subtypes in which JAK/STAT signal transduction is upregulated would respond the best to the proposed treatment.

We are aware that our results are preliminary and that the most important limitation of this report is the size of the group of patients with hematological malignancies, which did not allow for statistical analysis of the results. However, our data indicate that combining 1,25D analogs with a low concentration of Fludarabine is worthy of further investigation as a potential treatment strategy for older AML patients. Since the best pro-differentiation effect was observed after administration of PRI5202 to the blasts from MDS patients (Figure 8), we propose to further investigate the possible use of this analog in treatment of this disease. The upregulation of genes involved in antibacterial and anti-fungal defenses was observed also in blasts from AML patients (Figure 9), and when followed by protein expression, it may enhance patients’ ability to combat infections that commonly complicate the course of MDS or AML. Whether other leukemias and lymphomas respond to the combination of 1,25D analog with a low concentration of Fludarabine remains to be investigated. Importantly, Fludarabine, as well as some 1,25D analogs are already approved for clinical use in other conditions, which could facilitate a more rapid transition of this combination therapy to clinical trials, compared to entirely novel drugs.

## Data Availability

The raw RNAseq datasets for this study can be found in the Zenodo database [10.5281/zenodo.17609543; 10.5281/zenodo.1761026; 10.5281/zenodo.17610523; 10.5281/zenodo.17610718].

## Supplementary Materials

The following supporting information can be downloaded at: https://www.mdpi.com/article/10.3390/cells14231841/s1, Table S1: Primers used in RT-qPCR; Table S2: Characteristics of the donors involved in the study; Table S3: Numbers of genes regulated interactively by a combination of PRI5202 and Fludarabine; Table S4: The genes the most strongly upregulated by a combination of PRI5202 and Fludarabine in wt-937 cells; Figure S1: Gene alteration profile of selected genes in the FGFR and JAK-STAT pathways; Figure S2: Estimation of IC50 for Fludarabine in U937 cells; Figure S3: Viability of AML cells after exposure to PRI5202 or/and Fludarabine; Figure S4: Analysis of transcriptomes in AML cell lines exposed to PRI5202 or/and Fludarabine; Figure S5: Gating procedure for flow cytometry experiments on human bone marrow mononuclear cells; Figure S6: Non-viable cells in bone marrow samples exposed to PRI5202 or/and Fludarabine; Figure S7: Effects of FGFR overexpression, and response to Fludarabine in U937 cells.

## Abbreviations

The following abbreviations are used in this manuscript:

1,25D	1,25-dihydroxyvitamin D
AML	acute myeloid leukemia
APL	acute promyelocytic leukemia
AraC	cytarabine
AraCTP	AraC-triphosphate
ATO	arsenic trioxide
ATRA	all-trans retinoic acid
Bcl-2	b cell lymphoma 2 protein
*CAMP1*	gene encoding Cathelicidin Antimicrobial Peptide
CAPRI	calcium-promoted Ras inactivator
CMML	chronic myelomonocytic leukemia
*CYP24A1*	gene encoding Cytochrome P450 1,25D hydroxylase
DEGs	differentially expressed genes
DMSO	dimethyl sulphoxide
FGFR	fibroblast growth factor receptor
FLAG	Fludarabine, cytarabine, granulocyte colony-stimulating factor
FLAG-IDA	Fludarabine, cytarabine, granulocyte colony-stimulating factor, idarubicin
*FOP2-FGFR1*	fibroblast growth factor receptor oncogene partner 2–fibroblast growth factor receptor 1 fusion gene
GEF	guanine nucleotide exchange factor
HDAC2	histone deacetylase 2
*IDH2*	isocitrate dehydrogenase 2 gene
IFNγ	interferon γ
*ITGAM*	gene encoding CD11b
JAK	Janus kinase
MDS	myelodysplastic syndrome
*NPM1*	nucleophosmin gene
*PML*	promyelocytic leukemia gene
*RARA*	retinoic acid receptor α gene
RASA4	Ras p21 protein activator 4
RT-qPCR	reverse transcription quantitative polymerase chain reaction
*SLAMF1*	gene encoding CD150
SOCS	suppressors of cytokine signaling
STAT	signal transducer and activator of transcription
TEMED	N,N,N’,N’-tetra methylethylenediamine
TMM	trimmed mean of M values
*TREM1*	gene encoding CD354
VDR	vitamin D receptor
VDRE	vitamin D response element
