# Supplementary material for: Synergy in Immunostimulatory and Pro-Differentiation Effects of Vitamin D Analog and Fludarabine in Acute Myeloid Leukemias
Source: Cells. 2025 Nov 21;14(23):1841. doi: 10.3390/cells14231841 (PMC12691330; doi:10.3390/cells14231841)
Supplement: Supplementary file 1 [file cells-14-01841-s001.zip › cells-3936877-supplementary.pdf]

Supplementary Data

Supplementary Table S1: Primers used in RT-qPCR

No	Target gene	Primer sequence	Melting temperature (℃)	Reference
1.	*GAPDH*	Forward Primer:<br>5'-CATGAGAAGTATGACAACAGCCT-3′<br>Reverse Primer:<br>5'-AGTCCTTCCACGATACCAAAGT-3′	54.5	[20]
2.	*CYP24A1*	Forward Primer:<br>5′-CTCATGCTAAATACCCAGGTG-3′<br>Reverse Primer:<br>5′-TCGCTGGCAAAACGCGATGGG-3′	54.5	[20]
3.	*VDR*	Forward Primer:<br>5'-CCTTCACCATGGACGACATG-3'<br>Reverse Primer:<br>5'-CGGCTTTGGTCACGTCACT-3'	58.0	[41]
4.	*ITGAM*	Forward Primer:<br>5′-GGAACGCCATTGTCTGCTTTCG-3′<br>Reverse Primer:<br>5′-ATGCTGAGGTCATCCTGGCAGA-3′	60.4	https://www.origene.com
5.	*CD14*	Forward Primer:<br>5′- ACGCCAGAACCTTGTGAGC-3′<br>Reverse Primer:<br>5′- GCATGGATCTCCACCTCTACTG-3′	56.0	[42]
6.	*SLAMF1*	Forward Primer:<br>5′-ATGTTGCTGCCACAGAGCCTGT-3′<br>Reverse Primer:<br>5′-CCTTTGTTGGTCTCTGGTGTCAG-3′	60.0	https://www.origene.com
7.	*TREM1*	Forward Primer:<br>5′-CGATGTCTCCACTCCTGACTCT-3′<br>Reverse Primer:<br>5′-CAGCAAACAGGACAGAGAAGACC-3′	62.0	https://www.origene.com
8.	*CAMP*	Forward Primer:<br>5′-GACACAGCAGTCACCAGAGGAT-3′<br>Reverse Primer:<br>5′-TCACAACTGATGTCAAAGGAGCC-3′	60.4	https://www.origene.com
9.	*SOCS1*	Forward Primer:<br>5′-TTCGCCCTTAGCGTGAAGATGG-3′<br>Reverse Primer:<br>5′-TAGTGCTCCAGCAGCTCGAAGA-3′	60.4	https://www.origene.com
10.	*RASA4*	Forward Primer:<br>5′-GCTGAAGGACTTCATCACCAAGC-3′<br>Reverse Primer:<br>5′-TTGCCCTTGGTCCTGTGGATGA-3′	62.3	https://www.origene.com

Supplementary Table S2: Characteristics of the donors involved in the study

No.	Date of acquisition	Age	Sex	Disease	Fusion genes/oncogenes
1	06.02.24	77	M	MDS	nt
2	29.02.24	72	M	Healthy	nt
3	10.04.24	77	M	AML	NPM1 (+), FLT3-ITD (+), PML-RARA (-), RUNX1-RUNX1T1 (-), CBFB-MYH11 (-)
4	12.09.24	50	M	AML	CBF-MYH (+), FLT3-ITD (-), FLT3-TKD (-), NPM1 (-)
5	05.11.24	54	M	Healthy	nt
6	05.11.24	75	M	CMML	trisomy 19
7	07.11.24	61	F	MDS	nt
8	07.11.24	69	F	MDS	nt
9	13.11.24	35	M	AML	FLT3-ITD (+), NPM1 (+)
10	14.11.24	72	M	Healthy	nt
11	20.11.24	86	M	MDS	nt
12	16.12.24	78	M	Healthy	nt
13	19.12.24	58	F	AML	overexpression of EVI1
14	02.01.25	61	M	MDS	nt
15	02.04.25	61	F	MDS	nt
16	08.05.25	73	M	MDS	nt
17	06.06.25	68	M	healthy	nt

MDS – myelodysplastic syndrome; AML – acute myeloid leukemia; CMML - Chronic myelomonocytic leukemia; nt – not tested.

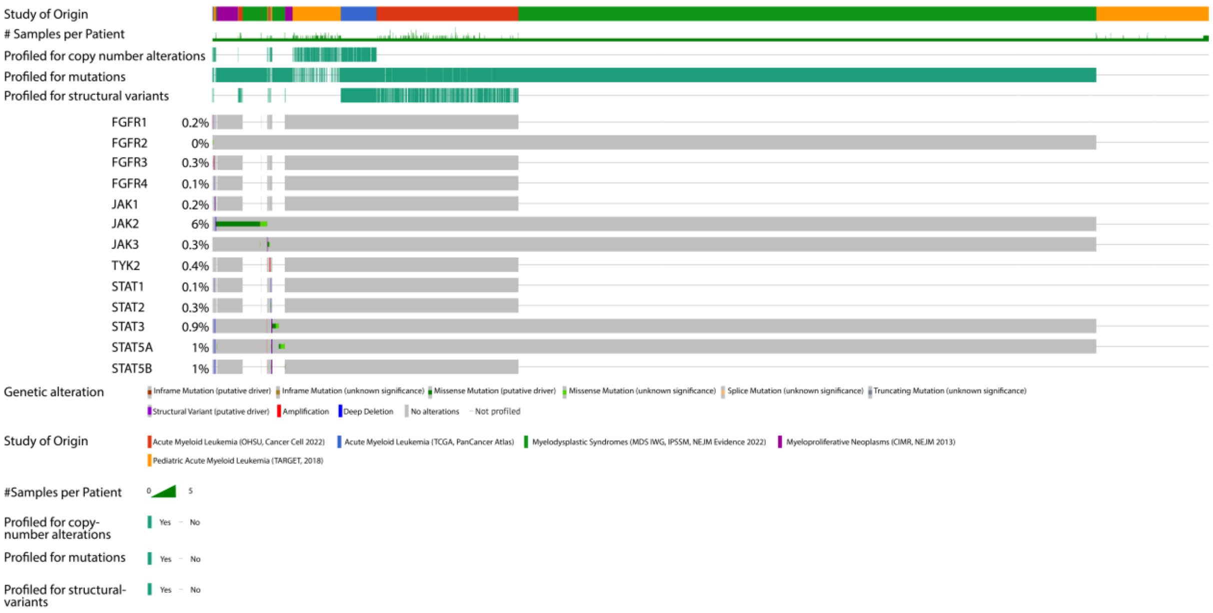

Supplementary Figure S1: Gene alteration profile of selected genes in the FGFR and JAK-STAT pathways. In order to analyze the frequency of genetic mutations occurring in various genes coding for proteins in the FGFR and JAK-STAT pathways (*FGFR1-4*, *JAK1-3*, *TYK2*, *STAT1-3*, *STAT5A-B*), the data from 6709 individuals (6946 samples) among

5 various studies (OHSU, Cancer Cell 2022; TARGET, GDC; TCGA, PanCancer Atlas; MDS IWG, IPSSM, NEJM Evidence 2022; CIMR, NJEM 2013) were obtained and processed in cBioPortal.

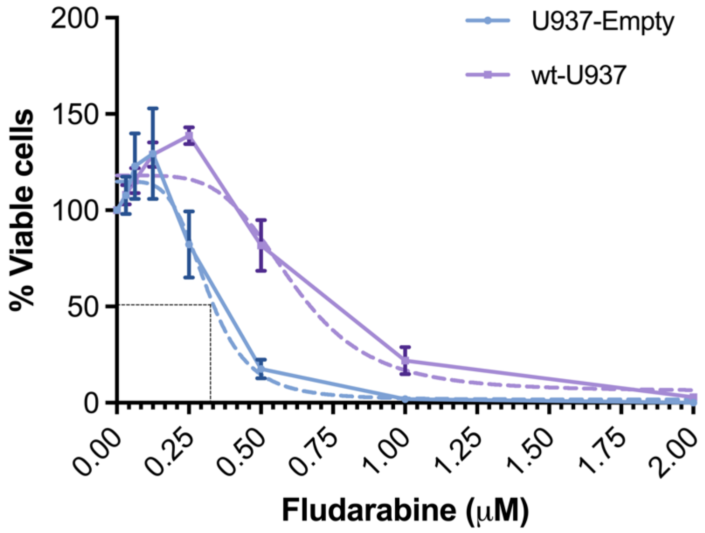

Supplementary Figure S2: Estimation of IC_{50} for Fludarabine in U937 cells. To determine the 50% inhibitory concentration (IC_{50}) against wt-U937 and U937-Empty cells in response to Fludarabine, the cells were exposed to increasing concentrations of the compound (0-2μM) for 96 hours, and an MTT-based cell viability assay was performed. Using GraphPad Prism 10 software, the four-parameter non-linear fit with variable slope was used to calculate the IC_{50} values for both cell lines. Wt-U937 and U937-Empty had IC_{50} of 610.2 nM and 315.5 nM, respectively, and all further experiments in this paper were performed with 315 nM of Fludarabine. The data are represented as mean ± SEM from 3 biological replicates.

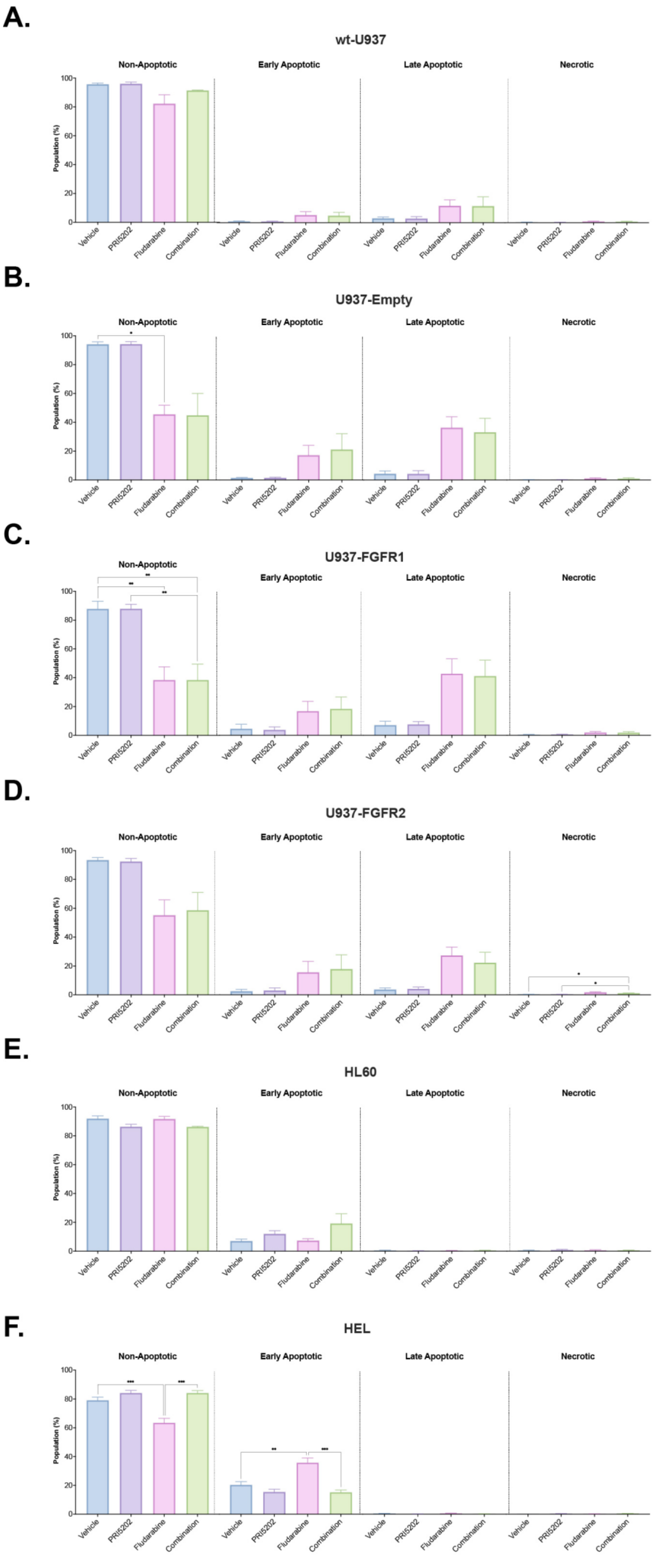
A.
wt-U937
B.
U937-Empty
C.
U937-FGFR1
D.
U937-FGFR2
E.
HL60
F.
HEL
Non-Apoptotic
Early Apoptotic
Late Apoptotic
Necrotic
Population (%)
Vehicle
PRI5202
Fludarabine
Combination

Supplementary Figure S3: Viability of AML cells after exposure to PRI5202 or/and Fludarabine. Wt-U937 (A), U937-Empty (B), U937-FGFR1 (C), U937-FGFR2 (D), HL60 (E) and HEL (F) cells were exposed to vehicle or to 10 nM PRI5202 or/and 315 nM Fludarabine for 96h, stained with Annexin V-APC and 7AAD from BioLegend according to manufacturer's instructions, and analyzed on Becton Dickinson Accuri C6 flow cytometer. The data are presented as mean ± SEM from at least 4 biological replicates. Significant differences are represented as * (p<0.033), ** (p<0.002) or *** (p<0.001).

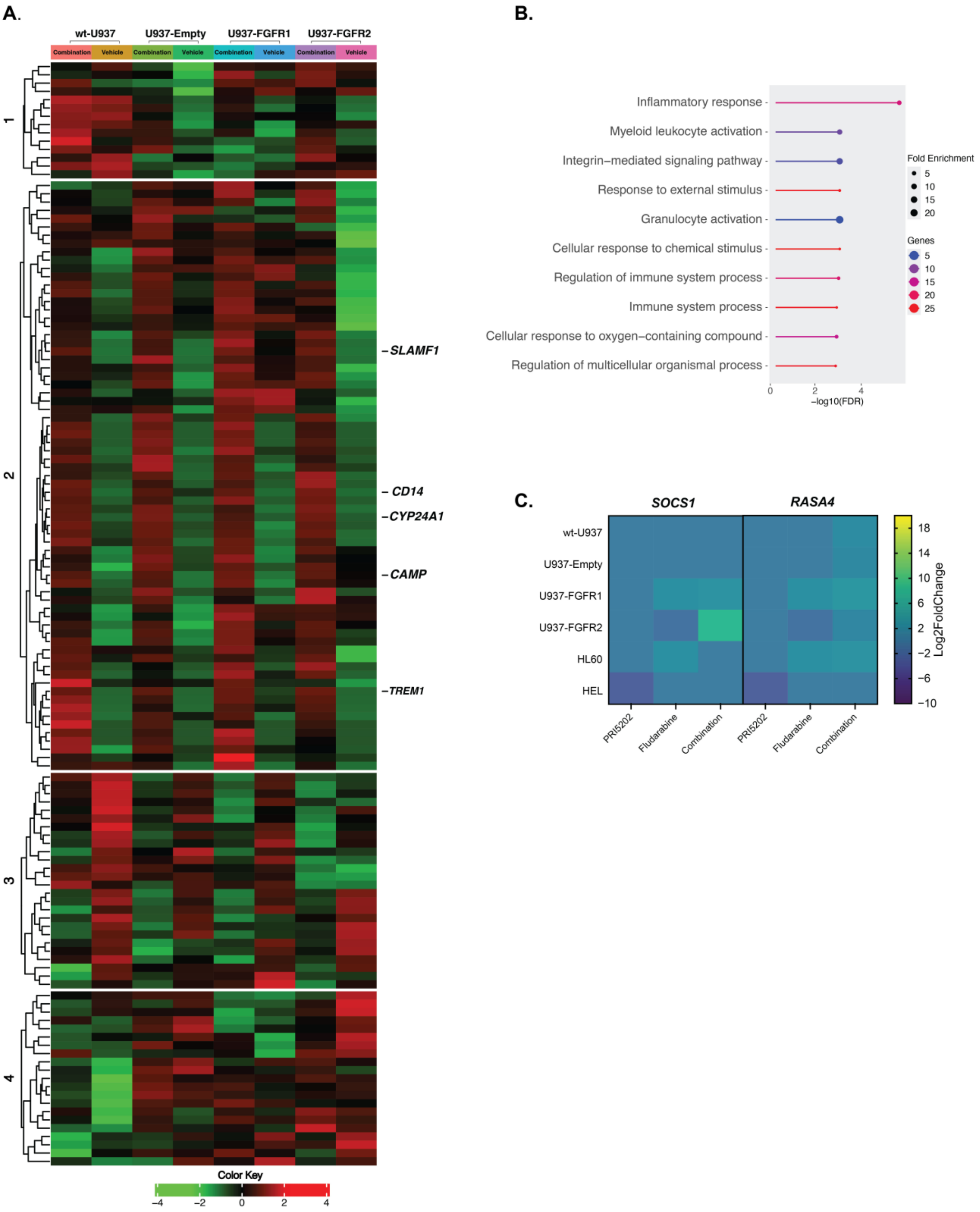

Supplementary Figure S4: Analysis of transcriptomes in AML cell lines exposed to PRI5202 or/and Fludarabine. Wt-U937, U937-Empty, U937-FGFR1, U937-FGFR2, HL60 and HEL cells were exposed to vehicle, or to 10 nM PRI5202 or/and 315 nM Fludarabine for 48 h and transcriptomic sequencing was performed. The initial DEG data were analyzed using NovoMagic software. Protein-coding DEGs were identified with $|Log_2FoldChange|$ of 1 and p-adj < 0.05. The DEGs whose expression levels enhanced synergistically after exposure to combination treatment were identified using formula (1). The genes whose expressions were upregulated synergistically in all U937 sublines were analyzed further by iDEP2.0 software, where the normalised read counts of genes in vehicle and combination-treated samples were uploaded. Using k-means clustering algorithm, the genes were ranked into 4 clusters post normalization and the clusters are shown (A). GO enrichment for biological processes of cluster 2 was performed to interpret the biological functions of the genes (B). Expression levels of *SOCS1* and *RASA4* with reference to vehicle control are presented as heatmaps (C).

Supplementary Table S3: Numbers of genes regulated interactively by a combination of PRI5202 and Fludarabine

Cell line	Gene regulation in response to a single drug	Synergistic effect in combination (n)	Antagonistic effect in combination(n)
**wt-U937**	Upregulated	773	564
**U937-Empty**	Upregulated	817	1128
**U937-FGFR1**	Upregulated	1094	1877
**U937-FGFR2**	Upregulated	1375	2241
**HL60**	Upregulated	537	932
**HEL**	Upregulated	646	1521
**wt-U937**	Downregulated	424	242
**U937-Empty**	Downregulated	236	769
**U937-FGFR1**	Downregulated	769	2626
**U937-FGFR2**	Downregulated	589	1093
**HL60**	Downregulated	161	1063
**HEL**	Downregulated	0	0

The gene expression was studied using the RNAseq method.

Supplementary Table S4: The genes the most strongly upregulated by a combination of PRI5202 and Fludarabine in wt-937 cells

Gene name	wt-U937	U937-Empty	U937-FGFR1	U937-FGFR2	HL60	HEL
CD14	15.6[1]	8.6	16	17	9.4	No synergy
CYP24A1	12	13.5	11.8	13	16.2	No synergy
CAMP	11.7	12.5	12.9	4.8	7	13.2
TREM1	11.3	11.9	7.7	12	6.2	No synergy
SLAMF1	10.4	10.7	5.1	11.3	No synergy	No synergy

[1] The numbers represent the $Log_2FoldChange$ of the genes synergistically upregulated by a combination of 10 nM PRI5202 and 315 nM Fludarabine, and sorted from the strongest to the weakest upregulation for wt-U937 cells. The gene expression was studied using the RNAseq method.

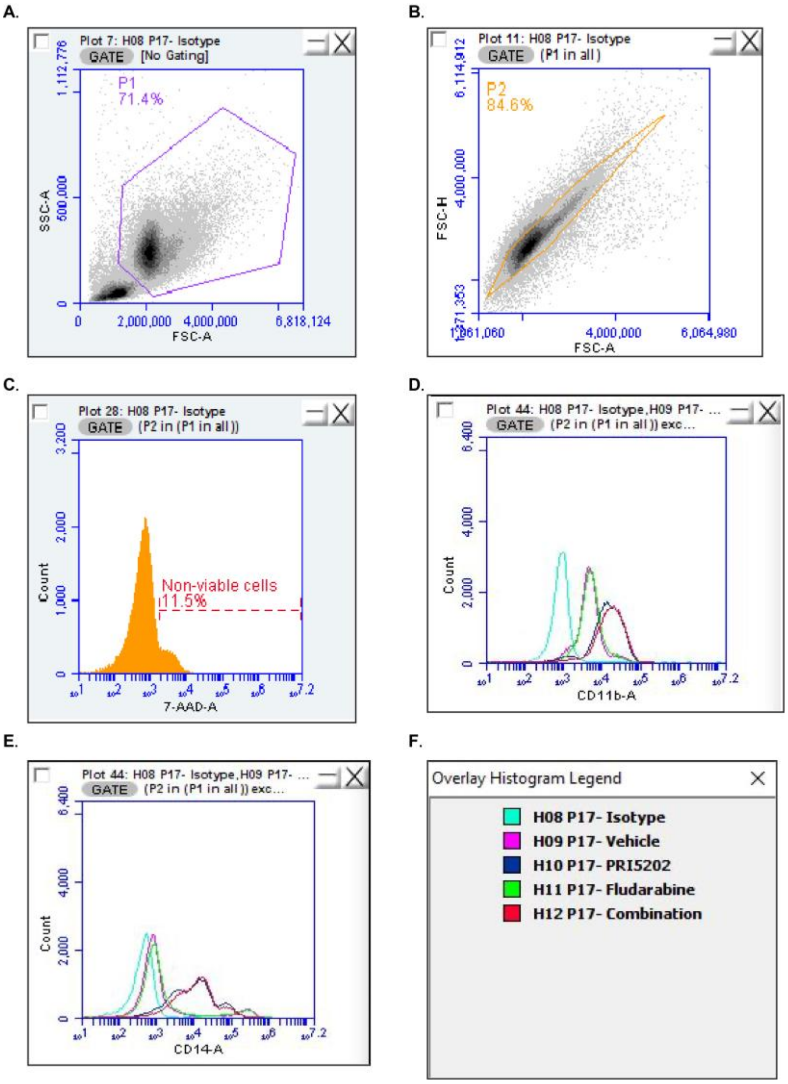

Supplementary Figure S5: Gating procedure for flow cytometry experiments on human bone marrow mononuclear cells. The first step in gating of the cells was based on forward and side scatter properties, where leukocytes except of lymphocytes were included in the P1 area (A). In the next step, from the population of leukocytes within the P1 gate, single cells were selected in the P2 gate using forward scatter area (FSC-A) vs forward scatter height (FSC-H) (B). Then the cells within the P2 gate were analyzed using a single parameter histogram for 7-AAD viability dye, and then the non-viable cells populations were gated out from further analysis (C). Examples of CD11b (D) and CD14 (E) surface marker expressions in viable, single leukocytes are presented according to the legend (F).

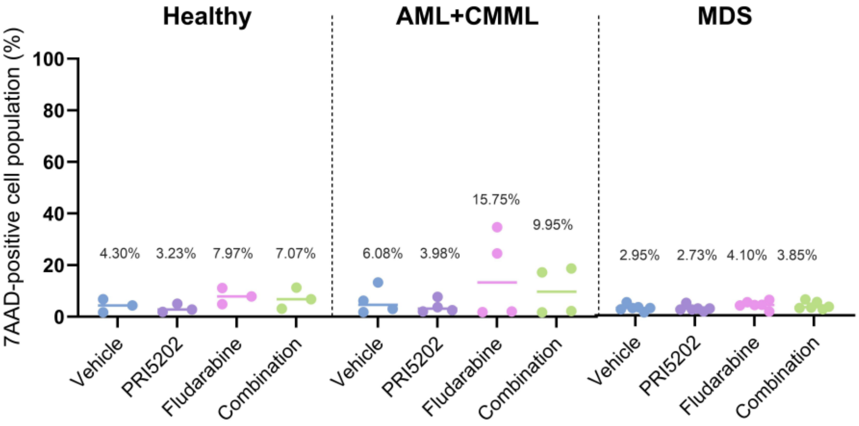

Supplementary Figure S6: Non-viable cells in bone marrow samples exposed to PRI5202 or/and Fludarabine. The bone marrow mononuclear cells obtained from human donors were exposed for 96 h to vehicle, or 10 nM PRI5202 or/and 315 nM Fludarabine. The percentages of non-viable cells (7AAD-positive) were measured in flow cytometry. The samples were categorized as MDS, AML+CMML or Healthy, according to the donor's diagnosis. Each dot represents the value obtained for a given sample, and the dash represents the mean value for the group. The mean values are presented above each graph.

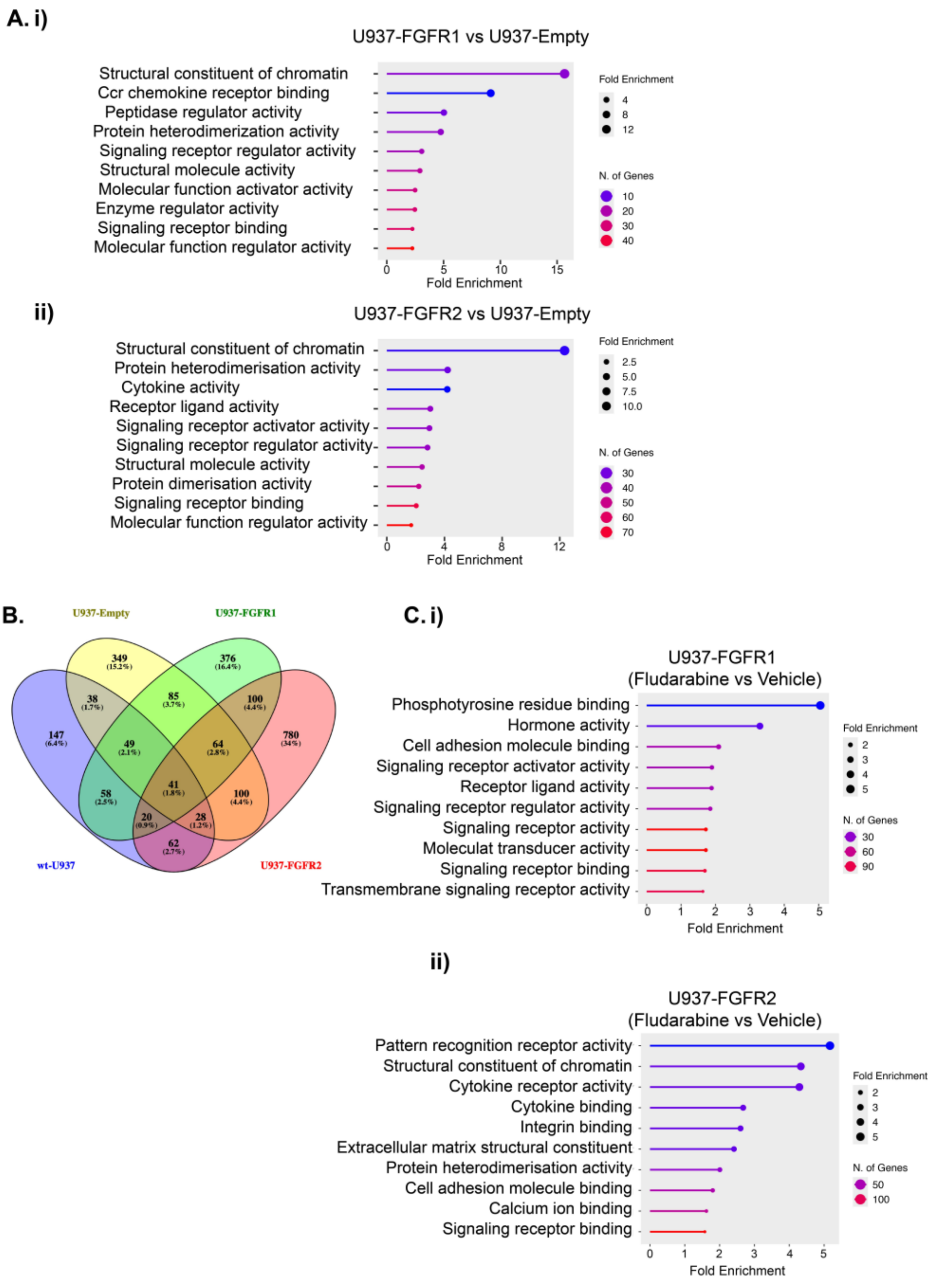

Supplementary Figure S7: Effects of FGFR overexpression, and response to Fludarabine in U937 cells. DEGs between U937-Empty and U937-FGFR1 (Ai) and between U937-Empty and U937-FGFR2 (Aii), with cutoff $|Log_2FoldChange|$ of 2 and adjusted p-value < 0.05, were analyzed for their molecular function by performing GO enrichment, and were sorted by the fold enrichment of the affected pathways. The influence of 48 h exposure to 315 nM Fludarabine (in comparison to vehicle-treated control samples) was analyzed in all U937 sublines. The numbers of genes differentially upregulated are presented as a Venn diagram (B). Their molecular functions were analyzed by GO enrichment for molecular functions, and sorted by the fold enrichment of the affected pathways, in U937-FGFR1 (Ci) and U937-FGFR2 (Cii).

References

20. Marchwicka, A.; Nowak, U.; Grembowska, A.; Jakuszak, A.; Poręba, P.; Marcinkowska, E. Overexpressed fibroblast growth factor receptors increase 1,25-dihydroxyvitamin D-dependent differentiation of acute myeloid leukemia cells. *Journal of Steroid Biochemistry and Molecular Biology* **2022**, *224*, 106173.
41. Gocek, E.; Marchwicka, A.; Baurska, H.; Chrobak, A.; Marcinkowska, E. Opposite regulation of vitamin D receptor by ATRA in AML cells susceptible and resistant to vitamin D-induced differentiation. *J Steroid Biochem Mol Biol* **2012**, *132*, 220-226.
42. Bock, C.; Kiskinis, E.; Verstappen, G.; Gu, H.; Boulting, G.; Smith, Z.D.; Ziller, M.; Croft, G.F.; Amoroso, M.W.; Oakley, D.H.; et al. Reference Maps of Human ES and iPS Cell Variation Enable High-Throughput Characterization of Pluripotent Cell Lines. *Cell* **2011**, *144*, 439-452, doi:https://doi.org/10.1016/j.cell.2010.12.032.
